# Developmental stage-dependent metabolic regulation during meiotic differentiation in budding yeast

**DOI:** 10.1186/s12915-014-0060-x

**Published:** 2014-09-02

**Authors:** Thomas Walther, Fabien Létisse, Lindsay Peyriga, Ceren Alkim, Yuchen Liu, Aurélie Lardenois, Hélène Martin-Yken, Jean-Charles Portais, Michael Primig, Jean Marie François

**Affiliations:** Université de Toulouse, INSA, UPS, INP, 135 Avenue de Rangueil, F-31077 Toulouse, France; INRA, UMR792 Ingénierie des Systèmes Biologiques et des Procédés, F-31400 Toulouse, France; CNRS, UMR5504, F-31400 Toulouse, France; Inserm U1085 IRSET, University of Rennes 1, Campus de Beaulieu, 35042 Rennes, France

**Keywords:** Differentiation, Flux analysis, Meiosis, Metabolic reprogramming, Metabolome, Systems biology, Transcriptome, Yeast

## Abstract

**Background:**

The meiotic developmental pathway in yeast enables both differentiation of vegetative cells into haploid spores that ensure long-term survival, and recombination of the parental DNA to create genetic diversity. Despite the importance of proper metabolic regulation for the supply of building blocks and energy, little is known about the reprogramming of central metabolic pathways in meiotically differentiating cells during passage through successive developmental stages.

**Results:**

Metabolic regulation during meiotic differentiation in budding yeast was analyzed by integrating information on genome-wide transcriptional activity, 26 enzymatic activities in the central metabolism, the dynamics of 67 metabolites, and a metabolic flux analysis at mid-stage meiosis. Analyses of mutants arresting sporulation at defined stages demonstrated that metabolic reprogramming is tightly controlled by the progression through the developmental pathway. The correlation between transcript levels and enzymatic activities in the central metabolism varies significantly in a developmental stage-dependent manner. The complete loss of phosphofructokinase activity at mid-stage meiosis enables a unique setup of the glycolytic pathway which facilitates carbon flux repartitioning into synthesis of spore wall precursors during the co-assimilation of glycogen and acetate. The need for correct homeostasis of purine nucleotides during the meiotic differentiation was demonstrated by the sporulation defect of the AMP deaminase mutant *amd1*, which exhibited hyper-accumulation of ATP accompanied by depletion of guanosine nucleotides.

**Conclusions:**

Our systems-level analysis shows that reprogramming of the central metabolism during the meiotic differentiation is controlled at different hierarchical levels to meet the metabolic and energetic needs at successive developmental stages.

**Electronic supplementary material:**

The online version of this article (doi:10.1186/s12915-014-0060-x) contains supplementary material, which is available to authorized users.

## Background

The meiotic developmental pathway in yeast enables both differentiation of vegetative cells into haploid spores that ensure long-term survival, and recombination of the parental DNA to create genetic diversity. Entry of *Saccharomyces cerevisiae* cells into meiotic differentiation is triggered in heterothallic diploid cells upon nitrogen starvation in the presence of a non-fermentable carbon source [1]. The genetic regulation of meiotic development was investigated in great detail. Studies employing single deletion mutants identified a set of more than 300 genes that are essential for the differentiation process [2-4]. Time-resolved transcriptome analyses identified meiotic regulation of approximately 1,600 protein-coding genes and 1,400 non-coding RNAs [5-8]. Key transcriptional regulators that orchestrate meiotic gene expression, that is, Ime1, Ume6, Sum1, and Ndt80 [9-12], are known, and their regulation depends on both environmental cues and completion of specific landmark events (reviewed in [13,14]). In addition, meiotic protein production is fine-tuned by transcript isoform-specific variations of translation efficiency [15].

Compared to the vast body of information available on the transcriptional machinery which governs the meiotic differentiation, rather little is known about the metabolic regulation of this process. Our knowledge is mainly limited to the following observations: sporulating cells experience strong changes of their macromolecular composition, characterized by the decrease of protein and RNA content [16], the accumulation of the reserve carbohydrates glycogen and trehalose [17], and the increase of the spore wall components chitin, mannan, glucan, and dityrosine [18,19]; acetate is assimilated via the cytosolic glyoxylate and the mitochondrial Krebs cycle, respectively [20]; concomitant with spore wall synthesis, glycogen is mobilized [17]; respiration is required throughout the whole differentiation process, whereas the presence of a carbon source becomes dispensable at later stages [21]; and defects in the glyoxylate cycle result for an unknown reason in aberrant spindle pole body formation and inhibition of the second meiotic division [22]. Proper co-regulation of metabolism with progression through a developmental program or a cell cycle is essential in terms of precursor and energy supply, and may also be important for creating an adequate intracellular environment to ensure cellular integrity [23]. Therefore, our study aimed at refining the analysis of metabolic regulation in the central metabolism taking place during meiotic differentiation using a systems-oriented approach that integrated transcriptome and metabolome data with high-throughput enzymatic measurements and the estimation of carbon flux repartitioning at mid-stage meiosis. As we shall show in this paper, the central metabolism in differentiating cells is regulated at different hierarchical levels, rendering conclusions drawn from transcriptomic data alone incomplete and highly deceptive.

We found strong developmental stage-dependent changes of the transcriptional activity of genes in the central carbohydrate metabolism and in the time course of 67 metabolites. Changes in the activity of 26 enzymes that function in the central carbon and nitrogen metabolism revealed meiosis-specific regulation of approximately half of the tested enzymes. The correlation between transcript levels and activities of most glycolytic enzymes varied strongly during progression through meiotic differentiation. Furthermore, meiosis-specific loss of glutamate dehydrogenase and phosphofructokinase activity, and glycogen mobilization correlated with the completion of meiotic landmark events. Finally, the need for correct homeostasis of purine nucleotides was demonstrated by the sporulation defect of the AMP deaminase mutant *amd1*, which exhibited hyper-accumulation of ATP that was accompanied by severe loss of guanosine nucleotides. Our study demonstrated that meiotic differentiation requires tightly controlled metabolic reprogramming that assures preservation of cellular energy levels and carbon and nitrogen flux repartitioning depending on the developmental stage of the cells.

## Results

Diploid heterothallic SK1 *MAT***a**/α cells were cultivated in YPA until mid-exponential growth phase. They were transferred to sporulation medium containing potassium acetate as the carbon source and trace amounts of nitrogen introduced by amino acid supplements that complemented the auxotrophies of the strain. Samples from at least two independent sporulation time courses were taken hourly until completion of meiotic differentiation for the measurement of genome-wide transcriptional activity, intracellular metabolite concentrations, and enzymatic activities. Our study focused on the regulation of the central metabolism that orchestrates carbon and nitrogen flux repartitioning and assures energetic homeostasis. Therefore, the transcriptome data released in our previous study [7] were re-analyzed with particular regard to the transcriptional activity of genes in glycolysis, pentose phosphate pathway (PPP), Krebs and glyoxylate cycle, gluconeogenesis, reserve carbohydrate metabolism, purine nucleotide metabolism, and glutamate metabolism. Accordingly, several enzymatic activities and metabolite pools located in these pathways were monitored, and a metabolic flux analysis was carried out at mid-stage meiosis. This allowed information on metabolic reprogramming to be inferred from four different datasets, and provided a high resolution analysis of metabolic reprogramming. To identify meiosis-specific metabolic regulation from the starvation response caused by the absence of nutrients, the behavior of the sporulating SK1 *MAT***a**/α strain was compared to a sporulation-deficient homothallic SK1 *MAT*α/α control strain cultivated in the same conditions. To minimize a potential impact of differing auxotrophic markers in both strains, all experiments were carried out using media with exactly the same composition, irrespective of the actual metabolic requirement of the strains for a particular supplement.

### Expression of genes implicated in central carbohydrate metabolism is meiotically regulated

**Figure 1:**
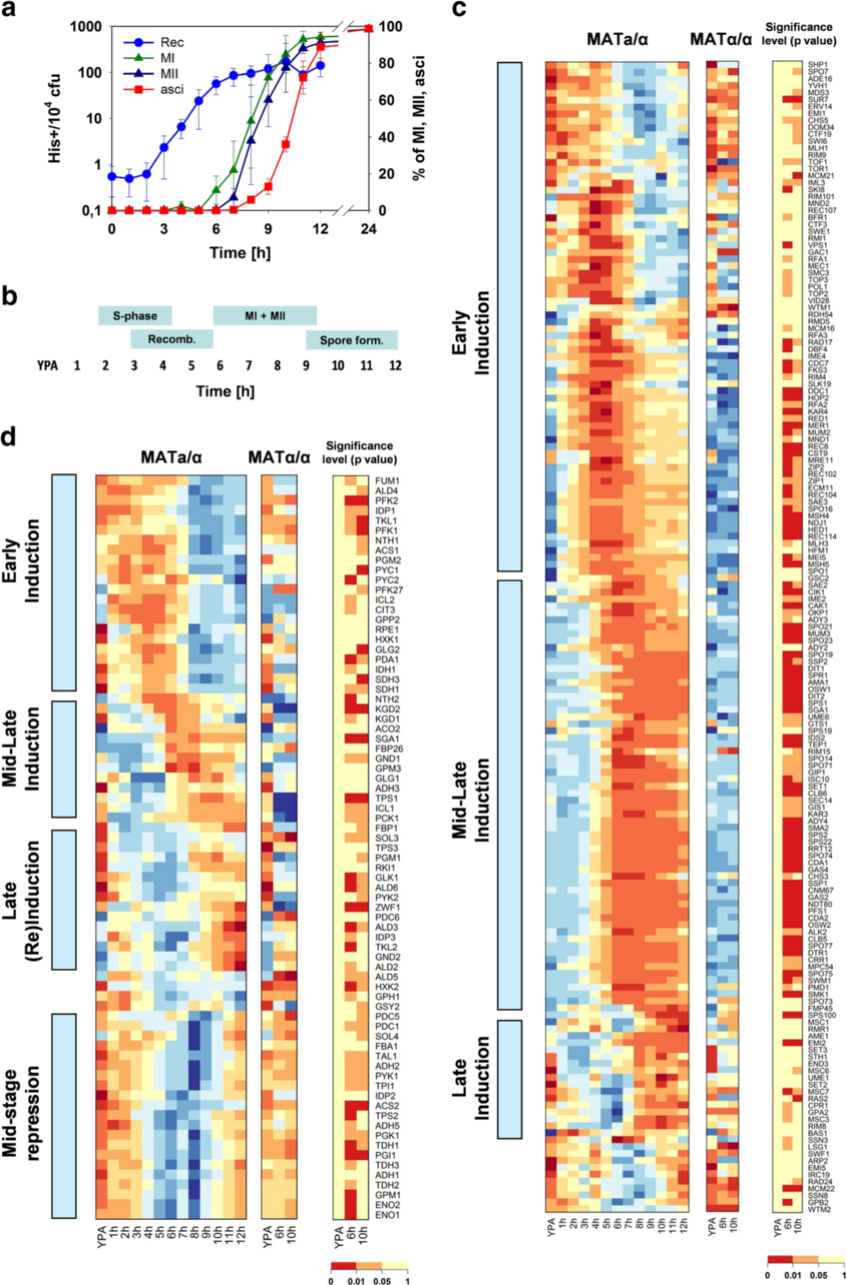
**Comparison of expression profiles of selected genes during meiotic development and progress in meiotic differentiation. (a)** Experimentally monitored and **(b)** schematically represented timing of meiotic landmark events. **(c, d)** Clustered gene expression patterns where rows correspond to a gene and columns correspond to time points after transfer from YPA to sporulation medium. Genes were clustered according to similar temporal expression patterns as indicated in the boxes at the left of each heatmap. The transcript levels in the sporulating SK1 *MAT* **a**/α strain were compared to the sporulation-deficient SK1 *MAT*α/α strain in YPA, and at 6 h and 10 h after transfer to sporulation medium. The significance levels of the observed differences between both strains were calculated in pairwise t-tests and are indicated next to panels that show expression levels. Genes with annotated meiotic functions are shown in C (GO terms: pre-meiotic DNA synthesis, reciprocal meiotic recombination, sporulation, ascus spore wall formation). Genes coding for enzymes in the central carbon metabolism are shown in **d**. Log2-scaled expression levels of each gene were scaled to have a mean of zero and a standard deviation of one. Each time point is colored according to signal strength ranging from low (blue), over medium (yellow), to high (red) expression. The expression data represent the average of two independent experiments. (Abbreviations: MI and MII denote the first and the second meiotic division, respectively; cfu = colony forming units; His+ = colonies that grow without histidine supplementation).

The SK1 *MAT***a**/α strain formed more than 80% spores during the first 12 hours after transfer to sporulation medium (Figure 1a). The characteristic landmarks of DNA replication, reciprocal recombination, meiotic divisions (MI/MII), and spore wall formation could be observed with little temporal overlap (Figure 1a,b) allowing us to study the physiological state associated with each of these different developmental stages. Genes with annotated functions in DNA replication, meiotic recombination, meiotic chromosome segregation, and spore wall formation exhibited transient induction patterns, with their time of peak expression associated with their function during meiotic differentiation (Figure 1c). This result is in agreement with earlier studies [5,6], and demonstrates that the temporarily confined expression patterns of meiotically regulated genes enables association of peak expression or repression with a specific developmental stage in our experimental setup.

We next analyzed the expression patterns of all the genes coding for the enzymes that build up the central carbon metabolism, that is, glycolysis (together with ethanol- and acetate-forming reactions), gluconeogenesis, PPP, Krebs and glyoxylate cycle, and glycogen and trehalose metabolism (102 genes, see Additional file 1). The expression level of 75 of these genes varied by at least two-fold over all time points during meiotic differentiation (Additional file 1). These genes were clustered according to similar temporal expression patterns during sporulation, demonstrating that the central carbon metabolism was subject to pronounced transcriptional changes during progression through meiotic differentiation (Figure 1d). To evaluate whether the observed changes were meiosis-specific, the expression levels of the 75 metabolic genes were compared between the sporulating SK1 *MAT***a**/α and the sporulation-deficient SK1 *MAT*α/α control strain at 6 h and 10 h after transfer to sporulation medium. We performed pairwise t-tests at the corresponding time points, wherein the standard deviations of transcript measurements of individual genes were estimated from the pooled SK1 *MAT***a**/α and SK1 *MAT*α/α replicates at the YPA time point (details of the statistical analysis are provided in Additional file 2; average relative standard deviation of transcript measurements was 8.5%). We found that the transcriptional activity of 59 genes was statistically different between both strains for at least one time point (Figure 1d), indicating that the observed transcriptional changes of central metabolic pathways were in large part meiotically controlled.

During early stages of meiotic development, when cells execute DNA synthesis and recombination (0 h to 5 h), genes functioning in the Krebs and glyoxylate cycle (*CIT3*, *IDP1*, *FUM1*, *ICL2*, *IDH1*, *SDH1*, *SDH3*, *ACS1*, *PDA1*), and in the upper part of glycolysis (*HXK1*, *PFK1*, *PFK2*, *PGM2*) exhibited a peak in their expression and became down-regulated during middle and late stages of sporulation. These observations are consistent with the high activity of the respiratory pathways and the priming of cells for the production of storage carbohydrates at the early stages of meiotic differentiation [16,17,20,21,24].

During middle sporulation, that is, during the meiotic divisions MI and MII (6 h to 8 h), genes encoding components of the 2-oxoglutarate dehydrogenase complex (Kgd; *KGD1*, *KGD2*) together with genes implicated in glycogen (*SGA1*) and trehalose (*NTH2*) mobilization, respectively, exhibited transient induction. Interestingly, expression of fructose-2,6-bisphosphatase (*FBP26*), the enzyme responsible for degradation of the allosteric activator of phosphofructokinase, fructose-2,6-bisphosphate, was also induced during this time and it maintained a high expression level until completion of spore formation. In addition, we identified a cluster of 21 genes whose expression levels were transiently repressed starting during recombination and reaching a minimum during the meiotic divisions (Figure 1d, mid-stage repression cluster), mainly comprising genes coding for glycolytic reactions (11 genes: *PGI1*, *FBA1*, *TPI1*, *TDH1*, *TDH2*, *TDH3*, *PGK1*, *GPM1*, *ENO1*, *ENO2*, *PYK1*) and ethanol-forming reactions (five genes: *ADH1*, *ADH2*, *ADH5*, *PDC1*, *PDC5*).

Together, these data point to the transcriptional activation of glycogen breakdown, the transient down-regulation of the entire glycolytic pathway, and re-modulation of Krebs and glyoxylate cycle activity at mid-stage meiosis. Visual comparison of the transcription profiles of the above listed metabolic genes with the earlier transcriptome study of Primig *et al*. [6] showed that their transcription patterns were highly similar in both studies. In particular, the meiosis-specific transcriptional down-regulation of the entire glycolytic pathway can also be observed in this earlier released dataset. However, Primig and colleagues analyzed the sporulation time course only until 10 h after transfer to sporulation medium, and their sampling frequency was lower than ours at later time points [6]. As a consequence, the late re-induction of the glycolytic genes and the transient nature of this transcriptional down-regulation was not captured by their work. In addition, the transcriptome analyses of the present study were carried out with two replicates (as compared to a single time course per strain analyzed by Chu *et al*. [5] and Primig *et al*. [6]), which enables statistical analysis of differences in transcript levels based on pairwise comparison of single time points (see below).

To identify factors that were implicated in the transient repression of glycolytic genes at mid-stage meiosis, all 5,348 differentially expressed genes (whose expression level varied at least two-fold within all conditions) were grouped into 10 clusters using the partitioning around medoids clustering algorithm (Additional file 3: Figure S1A). The expression pattern of the 585 genes in cluster six (Additional file 3: Figure S1A) showed mid-stage repression similar to the glycolytic genes mentioned above. Accordingly, 19 out of the 21 genes from the mid-stage repression cluster (Figure 1c) were found in this group of genes. Classification of mid-stage repressed genes according to Munich Information Center for Protein Sequences (MIPS) functional categories showed that genes with ribosomal (157) and glycolytic functions (11) and genes implicated in amino acid biosynthesis (5) were overrepresented (Additional file 3: Figure S1B). Hence, the gene cluster was strongly enriched for predicted targets [25] of the transcription factors (TFs) Sfp1, Fhl1, and Rap1, which are regulators of ribosomal genes [26-28], and of TFs regulating glycolytic genes, Gcr1 and Gcr2 [29], and amino acid biosynthesis, Gcn4 [30] (Additional file 3: Figure S1C). In addition, targets of the iron-responsive TF Yap5 [31] and the putative TF Stb4 [32] were overrepresented. When the 157 ribosomal genes were excluded from the TF analysis (note that 80% of these ribosomal genes were not differentially expressed in sporulating and sporulation-deficient cells, data not shown), only Gcr1, Gcr2, and Gcn4 targets were enriched among the remaining 428 genes.

Our data show that transcription of genes implicated in central metabolism is subject to strong temporal changes. In particular, the expression levels of a large number of glycolytic genes undergo a pronounced transient decrease suggesting down-regulation of the glycolytic pathway at mid-stage meiosis. The observations that targets of the glycolytic transcriptional regulators Gcr1 and Gcr1 were enriched in the mid-repressed gene cluster of sporulating cells (Additional file 4: Figure S2), that glycolytic genes were down-regulated only in sporulating cells (Figure 1d), and that expression of both Gcr1 and Gcr2 was significantly decreased at mid-stage meiosis only in sporulating cells (Additional file 4: Figure S2) argue for an implication of these TFs in the down-regulation of glycolytic genes. Interestingly, Fendt and co-workers [33] found that deletion of *GCR2* in cells that grow exponentially on glucose causes a decrease in the protein concentration of glycolytic enzymes without having an effect on the corresponding transcript levels. This result is somewhat counterintuitive and the reason for the apparent discrepancy with our data is not entirely clear. However, the experimental conditions applied in that study were very different from ours, and they did not investigate the effect of a parallel decrease of *GCR1* and *GCR2* transcription as it was found during decreased expression of glycolytic genes in our study.

### Transcript levels and corresponding enzymatic activities show strongly varying correlations during meiotic development

We next investigated how the observed changes in the expression of genes in the central metabolic pathways translate into modification of the corresponding enzymatic activities. Temporal changes were monitored in the enzymatic activities that build up glycolysis (together with ethanol-forming reactions), gluconeogenesis, Krebs and glyoxylate cycle, and the entry into the PPP. Because glutamate is at the heart of nitrogen metabolism, the activities of the catabolic and anabolic glutamate dehydrogenases were additionally quantified. While we tried to monitor all activities that comprise these pathways, our miniaturized high-throughput enzymatic assays did not yield reproducible results for the enzymes triosephosphate isomerase, succinate dehydrogenase, succinyl coenzyme A (CoA) synthetase, pyruvate dehydrogenase, and malic enzyme. They were therefore not considered in the analysis, which allowed us to cover 84% of the enzymatic activities located in the above mentioned pathways.

To test how enzymatic activities depend on transcript abundance, we calculated the ratio (*R(t)*) of the activity (*a*) of each enzyme over the sum of the transcript levels (*L*) of the homologous genes (*k*) encoding the enzyme (or the genes encoding the components of an enzymatic complex, for example, Kgd) according to Equation 1:

1 $$ R(t)=\frac{a(t)}{{\displaystyle {\sum}_{j=1}^k}{L}_j(t)} $$

*R(t)* was normalized to the corresponding ratio estimated in YPA (t0) to yield *R*_*N*_*(t)* (Equation 2):

2 $$ {R}_N(t)=\frac{R(t)}{R(t0)} $$

**Figure 2:**
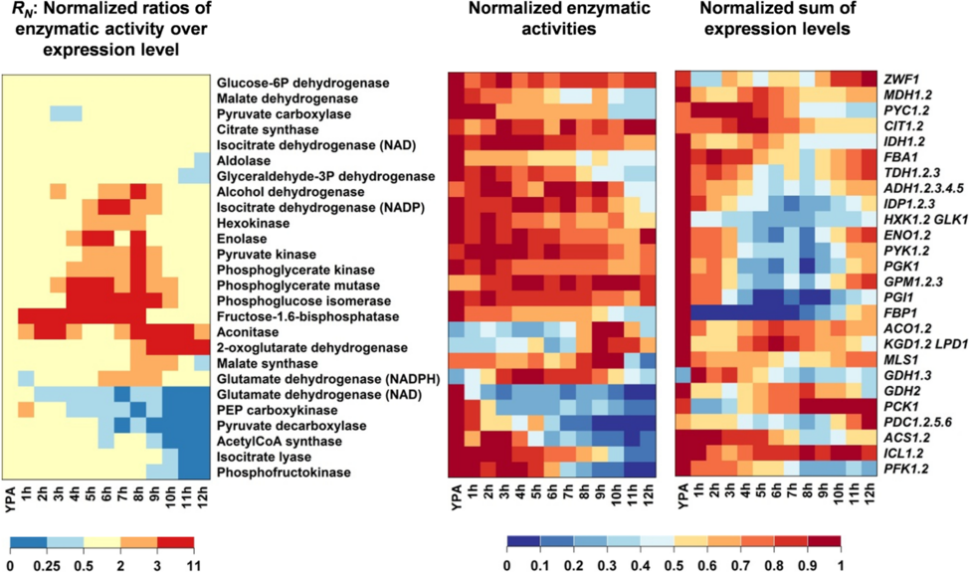
**Comparison of enzymatic activities and expression levels of the corresponding genes in sporulating cells.** Enzymes were grouped according to similar correlations between activity and transcription strength. Columns correspond to time points after transfer from YPA to sporulation medium. Each row corresponds to an enzymatic activity summarizing (left panel) the normalized ratio between enzymatic activity and the corresponding transcript levels, (middle panel) the normalized enzymatic activity, and (right panel) the normalized sum of the linearly scaled transcript levels. The ratios of activity over expression level were normalized to the corresponding ratio observed in YPA medium according to Equations 1 and 2. Expression levels and enzymatic activities were normalized to their maximum values observed over the whole time course. Data represent the average of at least two independent experiments.

This normalized value was used to visualize changes in the correlation between enzymatic activity and transcript abundance upon passage through the meiotic differentiation. For example, a value of *R*_*N*_ >1 indicates a higher enzymatic activity for a given transcript level than in the reference condition, which is exponential growth in YPA. The statistical significance of the observed changes was estimated by comparing the ratios of activity over the sum of expression levels on YPA (*R(t0)*) with the ratios at other time points (*R(t)*) in pairwise t-tests (details of the statistical analysis are provided in Additional file 2). We chose to show both *R*_*N*_ values and significance levels separately (Figure 2, Additional file 5: Figure S3A,B) because differences in *R*_*N*_ values that occurred in several neighboring time points may still bear biological relevance even though they were not statistically significant in pairwise t-tests that only considered individual time points.

The 26 enzymatic activities were grouped according to similar stage-dependent correlations between transcript abundance and corresponding activity (Figure 2). The statistical significance levels of the observed changes are provided in Additional file 5: Figure S3A. The activities of glucose-6-phosphate dehydrogenase, malate dehydrogenase), pyruvate carboxylase, citrate synthase, the NAD-dependent isocitrate dehydrogenase, fructose-1,6-bisphosphate aldolase, and glyceraldehyde-3-phosphate dehydrogenase largely followed the temporal changes of their transcripts (Figure 2). A highly variable correlation between transcript abundance and activity was found for the other monitored enzymes. The activities of the glycolytic enzymes hexokinase (Hxk), phosphoglucose isomerase (Pgi), phosphoglycerate kinase (Pgk), enolase (Eno), phosphoglycerate mutase (Gpm), as well as the NADP-dependent isocitrate dehydrogenase (Idp) and alcohol dehydrogenase (Adh) remained fairly constant during sporulation, whereas their transcript levels exhibited a pronounced transient decrease that started during recombination and reached a minimum during meiotic divisions. The strong drop in the transcription of fructose-1,6-bisphosphatase (Fbp) and NADPH-dependent glutamate dehydrogenase (Gdh1,3), occurring upon entry into meiosis and during recombination, respectively, was followed by decreasing activities only with a significant delay. Similarly, re-repression of the transiently induced genes encoding aconitase (Aco) and Kgd was not immediately followed by loss of enzymatic activity. Finally, the activities of NAD-dependent glutamate dehydrogenase (Gdh2), phosphoenolpyruvate carboxykinase (Pck), pyruvate decarboxylase (Pdc), acetyl-CoA synthase (Acs), isocitrate lyase (Icl), and phosphofructokinase (Pfk) decreased during sporulation and remained low even though transcription of the corresponding genes increased at later stages of meiotic development. The discussed changes in the correlations between activity and transcript abundance were statistically significant for all enzymes with the exception of Pdc.

The analysis of correlation between transcript abundance and enzymatic activity could not be carried out in the same detail for sporulation-deficient cells, because transcriptome data were only available for two time points in sporulation medium. However, when *R(t)* values at the 6 h and 10 h time points were analyzed in non-sporulating cells it became evident that the uncoupling of enzymatic activity and transcript abundance observed for several glycolytic enzymes (Hxk, Eno, Pgi, Pyk, Pgk, Gpm, Adh) at the meiotic M-phase did not occur in the control strain (Additional file 5: Figure S3B), providing evidence for a meiotically regulated phenomenon. By contrast, the correlations between activity and transcript levels of the other enzymes appeared to largely follow the same trends in both strains (Additional file 5: Figure S3B).

It became evident that an increase of enzymatic activity (Gdh1,3, Aco, Kgd) during progression through meiotic differentiation required induction of the corresponding genes. Conversely, most tested enzymatic activities followed a decrease of transcript abundance only with a strong delay (Fbp, Aco, Kgd), or even remained constant (Adh, Idp, Hxk, Eno, Pgk, Gpm, Pyk, Pgi). The lacking correlation between transcript abundance and enzymatic activity during specific stages of meiotic development may be explained by variations in the rates of mRNA translation and/or protein degradation. Indeed, a recent study showed that the translation efficiency strongly varies for a large fraction of mRNAs during meiotic development [15]. They found that the translation efficiency of the genes encoding the Fbp, Idp, and Gpm activities increased by more than three-fold during early and mid-stage meiosis compared to growth on YPA (Additional file 6, [15]). This may explain why these enzymes maintained a high activity despite their strongly decreased mRNA levels (Figure 2). In contrast, the translation efficiency of the enzymes Adh, Hxk Pyk, Pgk, Pgi, and Aco did not significantly increase at mid-stage meiosis (Additional file 6). Thus, a different mechanism must be responsible for the fact that their activities remained high (see discussion). Finally, the general decrease of translation efficiency at late stages [15] may explain why the activities of Pck, Pdc, Pfk, Acs, and Icl did not increase even though their transcripts exhibited late (re)induction (Figure 2).

In summary, enzymatic activities in the central metabolism are controlled by an enzyme-specific interplay of transcription strength, translational efficiency, and protein stability. In particular, the apparent down-regulation of the entire glycolytic pathway that was suggested by the strongly decreased transcription of the corresponding genes during mid-stage meiosis (Figure 1c) was not confirmed by enzymatic measurements. In contrast to the assumption of Ray and Ye [24], our data show that there is no direct link between transcriptional and enzymatic activity for most of the enzymes located in the analyzed central metabolic pathways.

### Enzymatic activities in the central metabolism exhibit strong meiosis-specific changes

**Figure 3:**
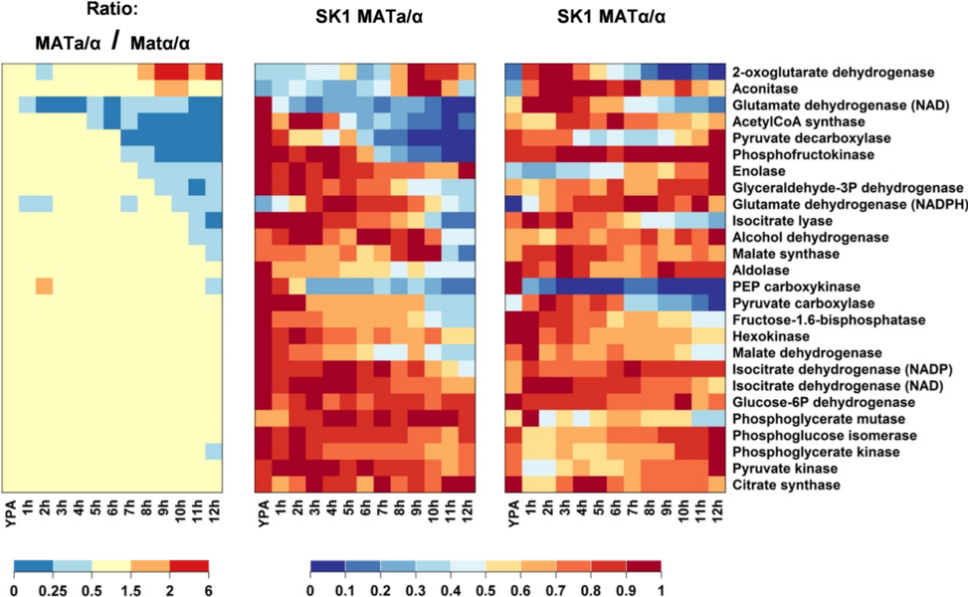
**Comparison of enzymatic activities in sporulating SK1** ***MAT*** **a/α cells and sporulation-deficient SK1** ***MAT*** **α/α cells after transfer to sporulation medium.** Enzymes were grouped according to similar correlations between their activities in the sporulating and the non-sporulating strain. Columns correspond to time points after transfer from YPA to sporulation medium. Each row corresponds to an enzymatic activity summarizing (left panel) the ratio between the enzymatic activity in sporulating and non-sporulating cells, (middle panel) the normalized enzymatic activity in sporulating cells, and (right panel) the normalized enzymatic activity in non-sporulating cells. Activities were normalized to their maximum value observed over the whole time course. The activities are provided in Additional file 8. Data represent the average of at least two independent experiments.

To assess which of the observed changes in enzymatic activities were specific for cells undergoing meiotic development, enzymatic measurements were compared to the sporulation-deficient SK1 MATα/α control strain cultivated under the same conditions (Figure 3 and Additional file 7: Figure S4, Additional file 8). Enzymes whose activities differed by more than two-fold between sporulating and control cells during at least two time points were considered to be regulated in a meiosis-specific manner. We found that 11 out of 26 enzymatic activities corresponded to this criterion (Figure 3): the increase in the activities of 2-oxoglutarate dehydrogenase and aconitase only occurred in sporulating cells; the decreasing activities of the anabolic NAD-dependent glutamate dehydrogenase, acetyl-CoA synthase, phosphofructokinase, pyruvate decarboxylase, glyceraldehyde dehydrogenase, isocitrate lyase, and alcohol dehydrogenase were only found in sporulating cells. The apparent meiosis-specific regulation of enolase was a particular case because this activity remained fairly constant in sporulating cells but experienced a strong increase in the starvation control. These differences were statistically significant except for pyruvate decarboxylase (Additional file 7: Figure S4). Likewise, malate synthase was a particular case: this enzymatic activity transiently peaked at M-phase in sporulating cells whereas it was highest at 3 h after transfer to sporulation medium in sporulation-deficient cells. It did not pass the above described fold-change criterion but the observed differences between the wild-type and the control strain were statistically significantly different over five consecutive time points, which argues for a meiosis-specific regulation of this enzyme.

The regulation of these enzymatic activities appeared to depend on progression through meiosis (Figure 3). The anabolic NAD-dependent glutamate dehydrogenase activity dropped immediately upon transfer to sporulation medium, and activities of acetyl-CoA synthetase and phosphofructokinase sharply decreased after completion of the early meiotic landmarks, that is, DNA replication and/or recombination. 2-oxoglutarate dehydrogenase and aconitase activities increased during entry into the meiotic divisions (compare to Figure 1a). By contrast, glyceraldehyde-3P dehydrogenase, pyruvate decarboxylase, isocitrate lyase, and alcohol dehydrogenase lost their activity rather gradually, making it difficult to associate a particular developmental stage with these changes.

Meiosis-specific regulation was observed for 11 out of the 26 tested enzymatic activities, which indicates that central metabolic pathways had to be remodeled to fulfill specific metabolic and energetic needs during different stages of meiotic differentiation. In particular, this remodeling included the nearly complete loss of phosphofructokinase and NAD-dependent glutamate dehydrogenase activity in differentiating cells points to altered carbon flux repartitioning via the glycolytic pathway, and meiosis-specific regulation of the glutamate metabolism, respectively. Furthermore, aconitase activity increased concomitantly with the induction of *ACO2*, which encodes the cytosolic isoform of this enzyme (Figure 1c, mid-late induction cluster). High activity of isocitrate lyase together with simultaneously increased activity of malate synthase and cytosolic Aco may point to an enhanced activity of the cytosolic glyoxylate shunt during meiotic M-phase and the onset of spore wall formation. Finally, the decreasing activity of acetyl-CoA synthetase limits the cell’s capacity to assimilate acetate, which is in accordance with the finding that extracellular acetate becomes dispensable during later stages of meiosis [21].

### Regulation of phosphofructokinase, NAD-dependent glutamate dehydrogenase, and storage carbohydrates depends on progression through meiotic development

**Figure 4:**
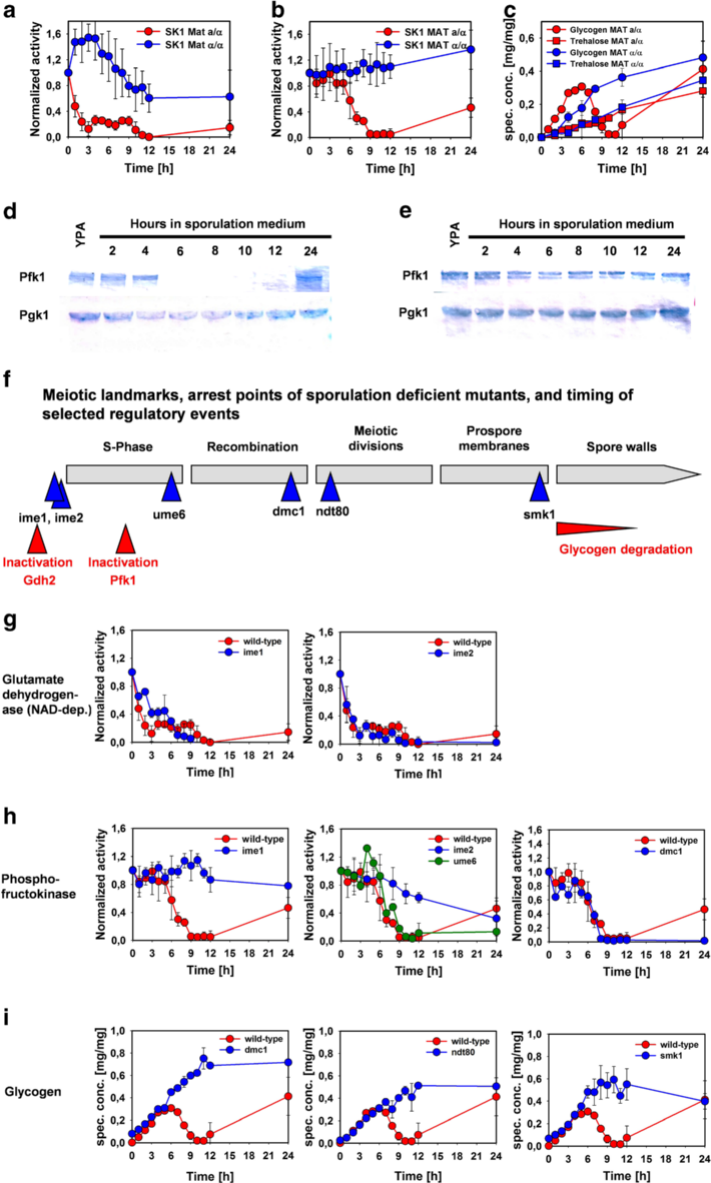
**Developmental stage-dependent regulation of metabolic events during meiotic development.** Meiosis-specific **(a)** decrease of glutamate dehydrogenase (NAD-dep.) activity, **(b)** inactivation of phosphofructokinase, and **(c)** glycogen degradation. **(d)** Western blot data for Pfk1 and Pgk1 in sporulating SK1 *MAT* **a**/α cells. **(e)** Western blot data for Pfk1 and Pgk1 in sporulation-deficient SK1 *MAT*α/α cells. **(f)** Timing of metabolic events depending on progression through meiosis inferred from the behavior of **(g)** glutamate dehydrogenase, **(h)** phosphofructokinase, and **(i)** glycogen in meiotic arrest mutants. Enzymatic activities were normalized to the activity in YPA. Data represent the average of at least two independent experiments.

The decrease of NAD-dependent glutamate dehydrogenase (Gdh2, Figure 4a) and phosphofructokinase (Pfk, Figure 4b) activities, as well as glycogen mobilization (Figure 4c), were specific for cells undergoing meiotic differentiation and did not occur in the sporulation-deficient control strain. Western blot analysis revealed that the decrease of Pfk activity was due to a meiosis-specific decrease of the protein’s concentration (Figure 4d), which did not occur in SK MATα/α control cells (Figure 4e). Using mutants that arrested meiotic development at well-defined stages (summarized in Figure 4f), we identified the landmarks where the decrease of the enzymatic activities and mobilization of glycogen were triggered. Cells deleted for *IME1* are unable to enter into meiotic development and arrest before the execution of meiotic DNA replication [9]. Similarly, the protein kinase Ime2 is required for the execution of early meiotic events [34,35]: depending on the strain background, *ime2* mutants are either defective or strongly delayed in meiotic DNA replication. In the *ime2* mutant used in this study, completion of DNA replication was delayed until 24 hours after transfer to sporulation medium (not shown). Cells defective in *UME6* require more time to complete DNA replication and arrest before meiotic recombination [10]. The mutant *dmc1* cannot exit from pachytene due to its inability for DNA double-strand break repair [36]. The TF Ndt80 is the master inducer of genes required for the meiotic divisions, and cells lacking the protein arrest at the onset of MI [11]. Finally, the protein kinase Smk1 is required for the synthesis of the outer spore wall layers and deletion mutants arrest after formation of the prospore membranes but before spore wall formation [37]. The decline of Gdh2 activity was neither prevented by the lack of *IME1* nor *IME2.* Thus, the decrease of Gdh2 activity is regulated by mechanisms that control entry into meiosis (Figure 4g). By contrast, loss of Pfk activity had an absolute requirement for functional *IME1* and was strongly delayed by the absence of *IME2*. However, it was unaffected by deletions in *UME6* or *DMC1* (Figure 4h). Therefore, it can be concluded that the decrease of Pfk was triggered upon entry into meiotic DNA synthesis, before execution of recombination. Glycogen degradation was absent in *dmc1*, *ndt80*, and *smk1* mutants, which caused previously unobserved accumulation of up to 70% glycogen (on dry mass basis, Figure 4i). Thus, glycogen degradation was triggered very late during sporulation and depended on completion of the meiotic divisions and prospore membrane formation. Contrary to earlier reports [38], substantial glycogen degradation still occurred in *sga1* mutants (Additional file 9: Figure S5). In addition, we found that trehalose accumulated in sporulating and starved cells (Figure 4c). However, this reserve carbohydrate was partially degraded in *dmc1* and *ndt80* mutants, but remained constant in the *smk1* strain (Additional file 9: Figure S5). Our data are also at variance with an earlier computational study which proposed that gluconeogenesis is completely turned off at late stages of meiotic development [24] whereas we find re-accumulation of glycogen in mature spores (Figure 4c). Taken together, these data demonstrate tight co-regulation of metabolic reprogramming and progression through meiosis, and hitherto unobserved partial mobilization of trehalose in response to specific arrest points in the developmental program.

### The metabolome in sporulating cells changes with progression through meiosis and differs strongly from starving cells

To identify further hotspots of metabolic regulation we measured intermediates of the above listed central metabolic pathways. Making use of the full power of our analytical systems, we additionally monitored metabolites located in purine and pyrimidine nucleotide metabolism, cell wall synthesis, and amino acid metabolism.

**Figure 5:**
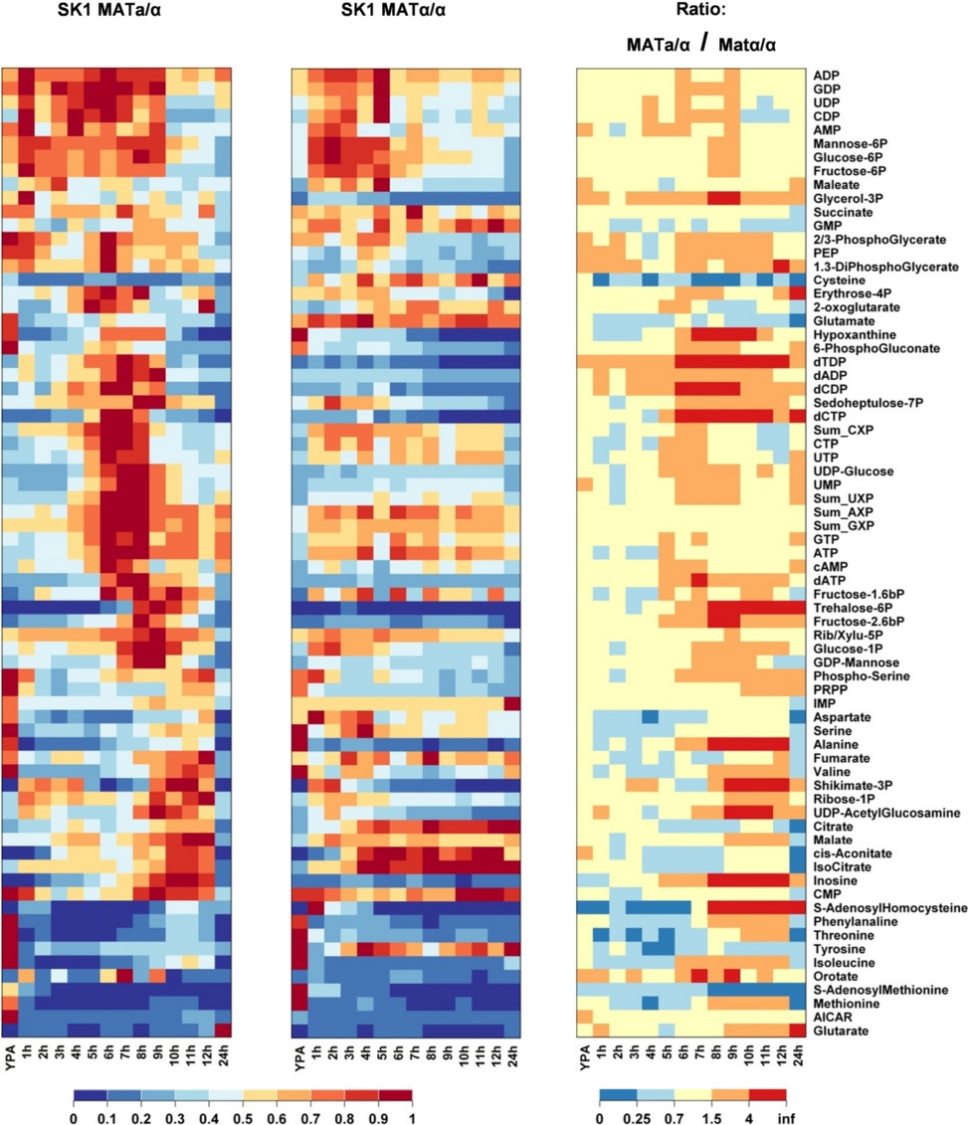
**Comparison of metabolite concentrations in sporulating SK1** ***MAT*** **a/α cells and sporulation-deficient SK1** ***MAT*** **α/α cells after transfer to sporulation medium.** Metabolites were clustered according to similar temporal concentration profiles in the SK1 *MAT* **a**/α cells. Columns correspond to time points after transfer from YPA to sporulation medium. Each row corresponds to a metabolite concentration summarizing (left panel) the normalized concentration in sporulating cells, (middle panel) the normalized concentration in non-sporulating cells, and (right panel) the ratio of the concentrations in sporulating and non-sporulating cells. Concentrations were normalized to the maximum values observed over the whole time course. Data represent the average of two independent experiments. Concentrations are provided in Additional file 11. (Abbreviations: Sum_AXP = [ATP] + [ADP] + [AMP] likewise for Sum_UXP, Sum_GXP, and Sum_CXP; Rib/Xylu-5P = pool of ribulose-5P and xylulose-5P).

Figure 5 displays the time course of the 67 monitored intracellular metabolite concentrations in sporulating and sporulation-deficient control cells after transfer to sporulation medium (Additional file 11). Strong temporal changes for nearly all intracellular metabolite pools were observed as the cells progressed through meiotic differentiation (out of the 67 measured metabolites, 66 varied more than two-fold, and 57 more than three-fold during sporulation, Additional file 10: Figure S6). These variations cannot be explained by the comparatively small changes in the cellular volume of the differentiating cells, which increases linearly by 45% until M-phase (6 h to 7 h) before it declines to yield tetrads that have nearly the size of the diploid mother cell [39]. Thus, the observed changes in metabolite concentrations indicate true metabolic rearrangements in meiotic cells.

In particular, we observed that glycolytic metabolites downstream of glyceraldehyde-3P dehydrogenase, namely phosphoenolpyruvate (PEP), 1,3diP-glycerate, and the 2/3P-glycerate pool (2- and 3-phosphoglycerate were measured as a single pool) displayed a transient decrease during meiotic DNA replication and increased upon exit from recombination and entry into M-phase. Similarly, erythrose-4P, 2-oxoglutarate, glutamate, 6-phosphogluconate, and hypoxanthine exhibited maximum concentrations during M-phase. Strikingly, also all (deoxy)nucleotide concentrations peaked at the exit from pachytene and the onset of M-phase (6 h to 8 h). This effect was due to an increase in both tri- and di-phosphates in the case of the deoxynucleotides, whereas the higher concentration of triphosphonucleotides was not accompanied by changes in mono- and diphosphorylated nucleotides. Trehalose-6P, fructose-2,6-bisP, the ribulose/ribose/xylulose-5P pool, glucose-1P and the cell wall precursor GDP-mannose peaked late in M-phase at the onset of spore formation. Most amino acids and Krebs and glyoxylate cycle metabolites behaved very similarly in differentiating cells exhibiting peak concentrations during spore wall formation. During this time, the spore wall precursors shikimate-3P and uridine diphosphate-N-acetylglucosamine, together with ribose-1P, inosine, CMP, and S-adenosylhomocysteine (SAH) also showed a strong increase in their concentrations.

To test whether individual metabolites were differentially regulated in the sporulating SK1 MAT**a**/α and the sporulation-deficient SK1 MATα/α strain, the differences in metabolite concentrations at individual time points were compared in pairwise t-tests in analogy with the above described statistical methods (Additional file 2). The average relative standard deviation for metabolite measurements was estimated at 24%. Significance levels for the observed differences between both strains are provided in Additional file 12: Figure S7. The metabolome of the sporulation-deficient control cells was also subject to strong variations (Additional file 10: Figure S6). However, the time course of most metabolite concentrations differed completely from sporulating cells (Figure 5). In particular, the accumulation of hypoxanthine, inosine, trehalose-6P, ribose-1P, deoxynucleotide triphosphates (dNTPs), shikimate-3P, uridine diphosphate-N-acetylglucosamine, alanine, and SAH was not observed in the starving SK1 MATα/α control strain, which points to hotspots of metabolic regulation in meiotic cells (Figure 5 and Additional file 12: Figure S7).

The increase of trehalose-6P (T6P) during meiotic M-phases is particularly interesting because this metabolite is a key glycolytic regulator whose exact function is still unclear [40,41], and whose presence is indispensable for yeast cells to grow under fermentative conditions. The T6P-synthetase encoding gene, *TPS1*, is also essential for meiotic development and its deletion was shown to prevent induction of the meiotic master-regulator Ime1 [42]. *TPS1* was classified as a mid-late induced gene in our study (Figure 1b). Its induction coincided with T6P production whereas transcription of the T6P phosphatase, which is encoded by *TPS2* (Figure 1c, mid-stage repression cluster), was transiently repressed during this time. Thus, T6P accumulation appears to be tightly controlled by differential induction of *TPS1* and *TPS2*. Our report showed for the first time that T6P does not only accumulate under fermentative conditions but also under fully aerobic conditions during meiotic development. Furthermore, the timing of *TPS1* induction and T6P accumulation (peak at mid-stage meiosis) does not correspond to the time of function of its apparent target, *IME1*, during the initiation of sporulation [42]. Both observations point to the need to revise the function of this key regulatory molecule. SAH is produced in methylation reactions that employ S-adenosylmethionine (SAM) as the methyl group donor. Therefore, depletion of SAM and meiosis-specific accumulation of SAH (Figure 5) are indicative for meiotic regulation of methylation reactions during M-phases and spore formation. Importantly, the accumulation of the purine salvage pathway metabolites inosine and hypoxanthine in differentiating cells points to the cell’s response to perturbations in their energy balance (see below, [43]). In line with this observation we detected accumulation of ribose-1P, which connects the purine salvage pathway with the PPP via the ribophosphomutase Rpm15 [44,45]. Transient accumulation of ribose-1P indicates Pnp1/Hpt1-catalyzed recycling of inosine into AXP (= [ATP] + [ADP] + [AMP]) [43,44].

Our metabolome analysis identified previously unknown metabolic rearrangements during execution of meiotic differentiation. In particular, we found that the metabolism of energy-bearing adenine nucleotides and accumulation of the key regulatory molecule T6P are differentially regulated in differentiating or starving cells.

### Proper regulation of AXP nucleotides is crucial for execution of meiotic differentiation

**Figure 6:**
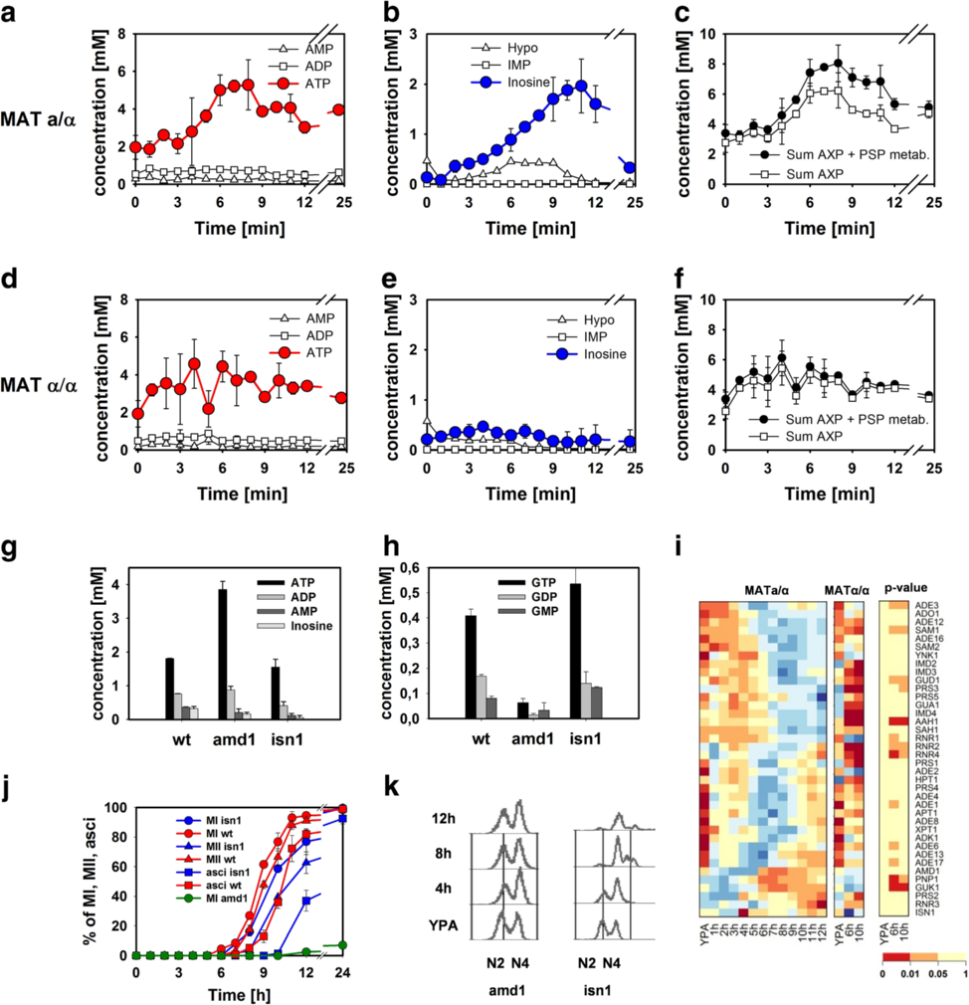
**Regulation of purine nucleotide metabolism in sporulating cells.** Concentrations of **(a, d)** adenosine nucleotides, **(b, e)** purine salvage metabolites, and **(c, f)** their sum in sporulating SK1 *MAT* **a**/α cells and sporulation-deficient SK1 *MAT*α/α cells after transfer to sporulation medium. Concentrations of **(g)** adenosine and **(h)** guanosine nucleotides in diploid wild-type, *amd1*- and *isn1*-mutant cells at 4 h after transfer to sporulation medium. **(i)** Expression of genes implicated in purine nucleotide synthesis and salvage in (left panel) sporulating and (middle panel) sporulation-deficient cells. Significance levels of differences in genes expression between both strains are shown in the right panel. Data were treated and presented as in Figure 1b and c. **(j)** Time course of chromosome segregation and spore formation in diploid wild-type, *amd1-* and *isn1-*mutant cells. **(k)** DNA content of diploid *isn1-* and *amd1*-mutant cells after transfer sporulation medium. (Abbreviations: AXP = [ATP] + [ADP] + [AMP]; Hypo = hypoxanthine; MI and MII denote the first and the second meiotic division, respectively; N2 and N4 denote diploid and tetraploid chromosome sets, respectively).

Metabolome analyses revealed a parallel increase of both ATP and inosine concentrations in sporulating cells (Figure 6a-c). While ATP also increased in the sporulation-deficient control strain, no inosine accumulation was observed in these cells (Figure 6d-f). Conversion of AXP nucleotides into inosine serves to maintain energetic equilibrium under transiently changing cultivation conditions [43] or in the presence of extracellular adenine [46]. Under mitotic conditions, AXP-to-inosine conversion is facilitated by the consecutive action of AMP deaminase, Amd1, and IMP-5′-nucleotidase, Isn1 [43]. To test whether inosine formation during meiotic development was catalyzed by the same system, diploid *amd1* and *isn1* mutants were transferred to sporulation medium and nucleotide pools and progression through meiosis was monitored. During growth on YPA, no differences in purine nucleotide levels were observed between wild-type and mutant strains (not shown). However, after 4 hours incubation in sporulation medium, *amd1* and *isn1* cells contained much less inosine compared to the wild-type (Figure 6g), confirming that Amd1 and Isn1 are the major enzymes catalyzing inosine formation.

Deletion of *AMD1* but not of *ISN1* entailed hyper-accumulation of ATP and concomitant depletion of the GXP (= [GTP] + [GDP] + [GMP]) nucleotide pool (Figure 6g,h). In addition to the strong deregulation of purine nucleotide metabolism, *amd1* cells were unable to sporulate and arrested during DNA replication (Figure 6j,k) while *isn1* cells exhibited a significant but comparatively mild slowdown of the differentiation program. We tested whether the sporulation defect of the *amd1* mutant was due to nitrogen limitation, which is normally released during the Amd1-catalyzed deamination of AMP. Addition of glutamate to the cultivation medium at a concentration that was still permissive for sporulation (0.3 g/L) allowed wild-type cells to form approximately 85% spores after 24 h, but did not rescue sporulation of *amd1* cells. Thus, nitrogen limitation was ruled out as the cause for impaired sporulation of this mutant.

Taken together, these results demonstrate that successful execution of meiotic development requires tight regulation of the AXP nucleotide pool. The major function of the Amd1/Isn1 enzyme system is to prevent hyper-accumulation of ATP, which causes depletion of the GXP pool and sporulation deficiency. By demonstrating that overproduction of ATP has fatal consequences, our results complement the analysis of Ray and Ye [24], who suggested that maximization of ATP production is the major objective of metabolic regulation in early sporulation.

The requirement for tight regulation of purine nucleotide metabolism is also reflected by developmental stage-dependent changes in the expression pattern of the corresponding genes. Genes implicated in purine nucleotide *de novo* synthesis were expressed during S-phase (0 h to 4 h), but became repressed at the same time when excess AXP nucleotides were sequestered into the inosine pool (5 h to 10 h) (Figure 6i). Accordingly, induction of *AMD1* was observed starting during middle sporulation. In agreement with the earlier transcriptome study of Primig *et al*. [6], these data indicate that purine nucleotide metabolism is at least in part transcriptionally regulated. However, most transcription levels of these genes were not statistically different between the sporulating and non-sporulating strain at the analyzed 6 h and 10 h time points (Figure 6i). This finding may in part be explained by the coarse temporal resolution of the transcriptome measurements for the SK1 *MATα/α* strain that did not allow a comparison of time points where peak expression of these genes occurred in sporulating cells. Nonetheless, it is likely that AXP and energy homeostasis is also subject to post-transcriptional (metabolic) regulation.

### Gluconeogenesis and assimilation of glycogen-derived glucose occur in parallel at mid-stage meiosis to facilitate spore wall synthesis

To characterize carbon flux repartitioning during co-assimilation of glycogen and acetate at mid-stage meiosis, a concentrated solution of ^13^C-fully labeled acetate ([U-^13^C] acetate) was added to two independent sporulating cultures that were pre-incubated for 7.5 h on 10 g/L unlabeled acetate. The cells were further incubated in medium in which approximately 60% of the acetate was fully labeled. Label incorporation was monitored by withdrawing samples from these cultures at regular intervals, and by measuring mass isotopologue distribution in 13 metabolite pools of the central metabolism by liquid chromatography coupled to tandem mass spectrometry (LC-MS/MS) [47] (average values are provided in Additional file 13).

The abundance (*A*) of labeled carbon in the metabolite pools was estimated using Equation 3:

3 $$ A={\displaystyle \sum_{i=0}^n}\frac{M(i)\cdot i}{n} $$

wherein *n* is the number of carbon atoms in the molecule and *M(i)* is the percentage of mass isotopologue (*i*). No complete mass isotopologue equilibrium was reached during the observed time span of 45 min. However, label incorporation in all metabolite pools started to saturate after 30 to 45 min, approaching the highest possible abundance of approximately 60% for several metabolites (succinate, PEP, 2/3P-glycerate). Because a compromise between the progression through differentiation and the observation interval had to be found, we based our calculations on the distribution of mass isotopologues at 45 min after addition of labeled acetate.

**Figure 7:**
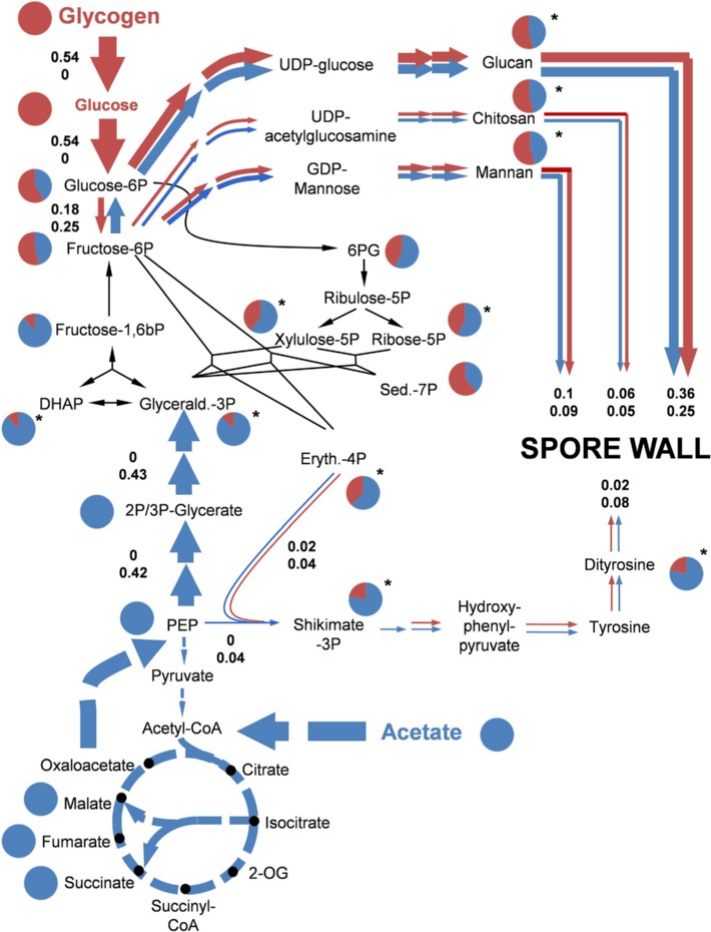
**Carbon flux repartitioning at mid-stage meiosis.** Pie charts show fractions of acetate-derived (blue) and glycogen-derived (red) carbon found in the metabolites. Asterisks indicate that these fractions were calculated (see Additional file 2). Metabolic flux analysis quantified the relative contribution of glycogen- and acetate-derived carbon to spore wall formation. (The fraction of acetate-derived carbon assimilated via Krebs and glyoxylate cycle was not quantified). Thickness of the arrows and depicted values correspond to the carbon flux over each reaction normalized to the overall carbon flux into spore wall formation (upper value = carbon flux derived from glycogen; lower value = carbon flux derived from acetate). Dashed and black lines indicate that no estimation of the exact flux can be provided. (Abbreviations: 6PG = 6P-gluconate, Sed-7P = sedoheptulose-7P, 2-OG = 2-oxoglutarate).

For several metabolites, the fraction of carbon atoms stemming from either glycogen or acetate was calculated based on label abundance and depicted in Figure 7 (for details see Additional file 2). Detection of acetate-derived carbon in the intermediates of glycolysis and the PPP indicated gluconeogenic activity at mid-stage meiosis. Furthermore, no glucose-derived carbon was detected in Krebs cycle metabolites when performing the reverse experiment, which consisted of adding low concentrations of ^13^C- fully labeled glucose to the sporulating cultures (not shown). These observations revealed that only acetate-derived carbon fuels the Krebs and glyoxylate cycles at this point in sporulation. To characterize how carbon flux stemming from acetate and glycogen converge in glycolysis and the PPP, we focused our analysis on carbon repartitioning in these pathways. The fraction of acetate-derived carbon decreased from nearly 100% in metabolites of the lower glycolysis (PEP, 2/3P-glycerate) to around 50% or even less in intermediates of the upper glycolysis (fructose-6P and glucose-6P) and the PPP (Figure 7). This observation can only be explained by strong dilution of the gluconeogenic flux in the upper glycolysis and the PPP caused by the assimilation of unlabeled carbon stemming from glycogen. Furthermore, the difference in the mass fraction of acetate-derived carbon between neighboring metabolite pools was highest between fructose-1,6bP (F16bP, 86%) and fructose-6P (F6P, 53%), indicating unidirectional gluconeogenic carbon flux at the F16bP/F6P node and confirming the absence of significant phosphofructokinase activity (see above). The distribution of mass isotopologues in both F16bP and F6P exhibited a major peak in the M + 3 fraction, which demonstrates significant gluconeogenic flux through aldolase and fructose-1,6bP-phosphatase (Fbp) (Additional file 2). However, in addition to the dilution of label, which occurred between F16bP and F6P, the ratio between different mass isotopologues also changed between both metabolites (Additional file 2). This observation argues for the implication of both the aldolase-Fbp route and the PPP in the catalysis of gluconeogenic flux. The estimation of the exact contribution of both pathways to gluconeogenic flux was not possible because our simulations were hampered by the fact that complete mass isotopologue equilibrium was not reached during the observed time span. However, our data show that at mid-stage meiosis, gluconeogenesis and glucose assimilation occur in parallel and identify F6P as the principal point in the metabolic network where glycogen and acetate-derived carbon fluxes converge.

At this stage in the developmental program, the synthesis of spore wall precursors represents the largest carbon sink apart from the formation of CO_2_ in the Krebs cycle [16]. The spore walls are composed of 55% glucan, 17% mannan, 9% chitosan, and 12% protein in which the shikimate-derived amino acids dityrosine, tyrosine, and phenylalanine account for roughly 60% of the carbon ([18], Additional file 2). The relative contribution of glycogen and acetate-derived carbon to the formation of the spore walls was estimated from these data and the label abundance in the precursors of the listed spore wall components (Figure 7, Additional file 2). It was found that glycogen and acetate almost equally contribute to the formation of spore walls (54% versus 46%, respectively) during the observed time span, which implies that the gluconeogenic flux departing from PEP (0.42) is of the same order of magnitude as the glycolytic flux departing from glycogen (0.53). Both carbon sources contribute almost equally to the formation of glucan, chitosan, and mannan, as inferred from the label abundance in the corresponding precursors glucose-6P and fructose-6P. Only in the dityrosine fraction of the spore wall, the calculated acetate-derived carbon fraction was approximately four times larger than from glycogen (Figure 7).

Taken together, our data show that at mid-stage meiosis glycogen breakdown and gluconeogenesis occur in parallel to assure repartitioning of acetate- and glycogen-derived carbon into spore wall synthesis. The fate of glycogen-derived carbon in cells undergoing meiotic differentiation is therefore different from carbon-limited cells, where glucose released from reserve carbohydrates undergoes complete glycolytic breakdown to eventually form ethanol. The strong decrease of Pfk activity in sporulating cells plays a key role in the regulation of this process because it blocks assimilation of glycogen-derived carbon through the glycolytic pathway. Our results are at variance with the computational metabolic analysis provided by Ray and Ye [24], who suggested that glycogen-derived carbon is funneled into glycolysis and respiratory metabolism thereby replacing acetate as the energy and carbon source. However, our metabolic flux analysis only covers the metabolic setup of sporulating cells at approximately 8 h of sporulation. Therefore, we cannot exclude that repartitioning of glycogen- and acetate-derived carbon, respectively, changes between spore wall synthesis and respiration at later time points.

## Discussion

### Metabolic regulation of central metabolism during meiosis relies on post-transcriptional control of enzymatic activities

For protein-coding genes involved in meiotic core functions - such as synaptonemal complex formation, recombination, entry into M-phase, and spore formation and maturation - strong correlation between timing of induction and time of function has been observed [1,5,6]. This notion does clearly not apply to the regulation of central metabolic pathways, where timing and strength of gene transcription did not correlate with enzymatic activity for most of the monitored enzymes. The relationship between the concentrations of mRNAs and their corresponding proteins is still not entirely understood. In large-scale proteomic studies, only roughly 40% of the changes in protein concentrations are explained by changes in their mRNA concentrations (see [48] for a recent review). For the enzymes monitored in our study, we found that the augmentation of activity required induction of the corresponding transcript while even strongly decreased transcript abundance did not, or only with a strong delay, cause a decrease in activity. Our analysis covered only a comparatively small set of enzymes, which does not allow us to draw general conclusions that go beyond the behavior of the enzymes that were actually investigated. However, our findings are in line with a report on the genome-wide correlation of transcript and protein abundance during exposure to osmotic stress [49]. Notably, these authors found that the transcriptional down-regulation of genes with GO-annotated metabolic functions only poorly correlated with their corresponding protein concentrations, which remained rather constant. In addition, a study on the regulation of glycolytic enzymes showed that their abundance was mainly determined by protein degradation and not transcription [50]. We complement this view by showing that in meiotically differentiating cells the strong repression of glycolytic genes has only a negligible impact on the catalytic capacity of this pathway.

For three (Fbp, Gpm, Idp) out of the eight enzymes that maintained their activity despite being transcriptionally down-regulated at mid-stage meiosis, a concomitantly increased translational activity was reported [15] that may explain why their activities remained fairly constant. This was not the case for the other five enzymes (Adh, Hxk, Pyk, Pgk, Pgi), thus pointing either to a low degradation rate of these proteins at mid-stage meiosis, or to a transient increase in their activity that might be brought about by post-translational modifications. Passage from one developmental stage to the other during sporulation occurs during comparatively short time spans, and the half-lives of glycolytic enzymes are significantly higher than 45 min [51]. Thus, it can be expected that the transient shut-down of the transcription of glycolytic enzymes does not significantly impact on the abundance of these proteins (which is particularly true in the absence of growth-dependent dilution), and leads the authors to prefer the hypothesis of a low degradation rate to explain the behavior of these enzymes. Following these lines of arguments, rapid depletion of an enzyme from the cells upon passage from one developmental stage to the other, as observed for Pfk, therefore requires an active degradation mechanism or must rely on a pronounced instability of the protein. Support for an active proteolytic degradation of Pfk can be derived from the observations that the protein is rapidly depleted from the cells and that cell cycle checkpoints exert tight control on its activity.

Another interesting question that remains is why the mRNA concentrations of glycolytic genes experienced such a pronounced transient drop in sporulating cells. Transiently decreasing the mRNA abundance of highly transcribed and stable proteins may reflect the cell’s adaptation to the absence of extracellular nitrogen, given that ammonia required for RNA and protein *de novo* synthesis has to be salvaged from dispensable cellular components. Alternatively, Lee and co-workers [49] suggested that reduction of normally abundant transcripts during massive induction of newly synthesized proteins, as observed during the adaptation to stress or upon passage from one developmental stage to the other, may reflect the cell’s need to free some of its translational machinery for the synthesis of new proteins. While we cannot provide a conclusive answer to this question, our data clearly indicate that enzymatic activities in central metabolism are subject to pronounced post-transcriptional regulation during meiotic differentiation, rendering the inference of information on metabolic regulation from transcriptome data alone highly deceptive.

### Carbon flux repartitioning into spore wall precursors is orchestrated at the level of phosphofructokinase

In an earlier study of Dickinson *et al*. [20] on the carbon flux repartitioning in sporulating cells, it was found that glyoxylate shunt and the Krebs cycle function in parallel to provide glutamate and PEP for gluconeogenesis. In addition, they reported that the ^13^C labeling pattern in trehalose was consistent with a significant contribution of the PPP to gluconeogenic flux. However, their work did not provide any information that is required to relate the temporal changes in the labeling patterns with a particular developmental stage; neither had they reported sporulation efficiencies of their experiments that are necessary to assess the relevance of their results. In addition, because sporulating cells were incubated on labeled acetate throughout the experiment, they could not capture carbon flux rearrangements that were due to the release of glycogen at mid-stage meiosis.

Using our experimental procedure it was possible to demonstrate that acetate and glycogen are assimilated in parallel during spore wall synthesis (that is, at approximately 8 h after transfer to sporulation medium). In particular, we showed that the - and the glycogen-derived carbon fluxes converge in the glycolytic pathway at the level of fructose-6P, from where they are further channeled into the synthesis of spore wall precursors. This unique functional setup of glycolysis requires subtle control. We have shown by direct measurements that the activities of the glycolytic pathway enzymes remain largely constant during the switch from the accumulation to the breakdown of glycogen. Thus, the regulation of carbon flux repartitioning into spore wall synthesis relies almost exclusively on the inactivation of Pfk that occurs at this stage.

In earlier studies on glycolytic regulation it was found that Pfk activity varies by at most a factor of two between fully fermentative and gluconeogenic conditions [50,52]. Even under fully gluconeogenic conditions, Pfk activity is more than 20 times higher than Fbp (see, for example, our data for SK1 *MATα/α*, Additional file 8). Thus, it is clear that the direction of net-flux across the F16bP/F6P node is determined by allosteric control of the Pfk activity [53] in proliferating or resting cells. Moreover, periodic breakdown of reserve carbohydrates during carbon-limited respiratory growth commonly causes formation of ethanol, which indicates glycolytic breakdown of glucose [54-56]. Thus, mere allosteric control of Pfk is likely to be insufficient when glucose assimilation during meiotic development will be halted at fructose-6P. The complete loss of Pfk activity, however, prevents glycolytic assimilation of glycogen-derived carbon and ensures unidirectional gluconeogenic flux at the F16bP/F6P node.

It is of note that Pfk is by far the best-studied glycolytic enzyme and subject to subtle allosteric control by several metabolites [53]. The previously unobserved complete depletion of the phosphofructokinase protein from sporulating cells adds another mechanism that controls the activity of this key glycolytic enzyme.

### Nitrogen salvage for spore wall formation requires tight regulation of glutamate and 2-oxoglutarate

Glutamate is at the heart of the metabolism of amino acids and spore wall components, because it participates in almost all transamination reactions with proteinogenic amino acids and functions as the principal amino group donor for production of the spore wall components tyrosine and chitin (via glutamine). Regulation of glutamate availability is therefore of critical importance for meiotic development. Transient accumulation of glutamate was reported in sporulating cells [1,20] and suggested to be controlled by an observed decrease of 2-oxoglutarate dehydrogenase (Kgd) activity [57]. We also found that glutamate transiently increased in sporulating cells (Additional file 14: Figure S8A). However, we did not detect a significant decrease of Kgd activity at the onset of sporulation but found that the transient glutamate accumulation coincided with the activation of anabolic glutamate dehydrogenase, Gdh1, and parallel inactivation of the catabolic glutamate dehydrogenase, Gdh2 (Additional file 14: Figure S8A,C), which argues for a major role for these two enzymes in controlling the glutamate pool.

In agreement with an earlier study by Betz and Weiser [58], we found that net degradation of intracellular protein occurs in sporulating cells (Additional file 14: Figure S8B). We complement their analysis by showing that this protein degradation in the sporulating SK1 *MAT***a**/α strain first sets in at the onset of the meiotic divisions and lasts until completion of spore formation. This suggests that the nitrogen released upon protein degradation is specifically required for the synthesis of the spore wall components chitosan and dityrosine.

In order to maintain the thermodynamic capacity of glutamate to serve as amino group donor for tyrosine production, accumulation of 2-oxoglutarate (2-OG) has to be prevented. Indeed, the pronounced drop of glutamate concentration during spore formation is not accompanied by an increase of 2-OG, but coincides with a strong degradation of this metabolite, whose concentration reaches a minimum at 9 h to 10 h (Figure 4 and Additional file 14: Figure S8C). Our analysis suggests that the decrease of 2-OG is mainly facilitated by activation of Kgd, which assures degradation of 2-OG (Additional file 14: Figure S8C). In addition, high isocitrate lyase activity together with the parallel activation of cytosolic aconitase and malate synthase (Figure 2 and Additional file 14: Figure S8C) might be indicative of an increased capacity of the glyoxylate shunt to accommodate carbon flux, thereby creating the potential to bypass 2-OG production in the Krebs cycle.

In summary, successful meiotic differentiation in yeast requires salvage of nitrogen from dispensable cell components and its redistribution into *de novo* synthesis of proteins, mRNA, and the spore wall. Therefore, the central metabolite in nitrogen metabolism, glutamate, and its precursor 2-OG are tightly regulated in a developmental stage-dependent fashion.

### Homeostasis of cellular energy levels is actively controlled

Our data demonstrated that the function of Amd1 and Isn1 ensured purine nucleotide homeostasis and wild-type sporulation dynamics. Interestingly, inosine and ATP accumulated in parallel in sporulating cells (Figure 5a-c), in contrast to inosine accumulation upon decreasing ATP levels in fermenting cells [43]. However, it was recently reported that lack of Amd1 together with the presence of extracellular adenine causes unscheduled accumulation of ATP and depletion of the guanosine nucleotide pool. This behavior was explained by the repression of *de novo* nucleotide synthesis by adenine, and the incapacity of *amd1*-mutant cells to convert this purine base into GXP nucleotides [46]. It is conceivable that a similar deregulation of the *de novo* synthesis of purine nucleotides caused accumulation of ATP and depletion of GXPs in sporulating cells. This raises the question of the origin of ‘excess’ adenosine nucleotides and bases, respectively. Our metabolome analyses revealed a significant decrease of SAM after transfer of the cells to sporulation medium that was not accompanied by an equimolar increase of SAH. Demethylation of SAM gives rise to SAH, whose subsequent hydrolysis by S-adenosylhomocysteine hydrolase, Sah1, produces homocysteine and adenosine. Given that adenosine is readily converted into AMP by adenosine kinase, Ado1, the release of adenosine from SAH is very likely to be the cause for the observed imbalance of AXP nucleotides. In addition, earlier studies reported pronounced RNA degradation in sporulating cells that starts after completion of recombination [1]. Both phenomena may provide an explanation for the accumulation of intracellular adenosine nucleotides. In any case, our study provides evidence that active control of metabolic energy levels is crucial for successful completion of meiotic differentiation.

## Conclusions

Our study provides a system-level analysis of metabolic regulation during meiotic differentiation in yeast. We show that the physiological state of meiotic cells is dynamically remodeled depending on the progression through the developmental program. The metabolic reprogramming occurs at different hierarchical levels, which renders this process highly complex. We have uncovered numerous metabolic events that are unique for meiotically differentiating cells. These findings will guide further research in yeast and higher organisms to understand how proper metabolic regulation provides the basis for successful completion of meiotic development.

## Methods

### Strains and cultivation conditions

We used wild-type and mutant derivatives of the *S. cerevisiae* SK1 strain (Additional file 2: Table S1). Sporulation experiments were carried out as described and reproducibility of sporulation kinetics was verified by monitoring characteristic landmark events [7]. Briefly, pre-cultures were grown at 30°C to mid-exponential phase in YPA medium (5 g/L yeast extract, 10 g/L bacto peptone, 1.7 g/L yeast nitrogen base, 10 g/L potassium acetate, 5 g/L ammonium sulfate, 5.1 g/L potassium phthalate, pH 5.5), shaken at 200 rpm on rotary shakers (Infors) until cell density reached 2.6°10^6^ cells/mL. Cells were harvested by centrifugation (5000 g, 4 min, Beckmann), washed with sterile water, and inoculated into sporulation medium (20 g/L potassium acetate supplemented with lysine, uracil, arginine, histidine, and leucine, 50 mg/L each, to complement auxotrophies of the used strains) at a cell density of 2 × 10^6^ cells/mL and incubated at 200 rpm and 30°C. All sporulation and growth experiments were carried out at a ratio of culture liquid to total flask volume of 1:5.

### Transcriptome, transcription factor, and gene ontology term analyses

Raw transcriptome data were published in an earlier study [7]. They are available via the European Bioinformatics Institute’s repository ArrayExpress [59] [accession no. E-TABM-915 (*MAT***a**/α and *MAT*α/α)].

In our TF analysis we referred to the dataset provided by MacIsaac *et al*. [25] who identified associations between TFs and genes based on the TF binding motif and its conservation among different yeast species (supplementary data of [25], file ‘TF_binding_p0001_cons1.txt’). To identify enrichment of TF targets among a given gene cluster, a hyper-geometric test was performed according to [60] using Equation 4:

4 $$ p=1-{\displaystyle \sum_{i=0}^{k-1}}\frac{\left(\begin{array}{c}\hfill M\hfill \\ {}\hfill i\hfill \end{array}\right)\left(\begin{array}{c}\hfill N-M\hfill \\ {}\hfill n-i\hfill \end{array}\right)}{\left(\begin{array}{c}\hfill N\hfill \\ {}\hfill n\hfill \end{array}\right)} $$

wherein *N* is the total number of genes, *M* is the number of annotated targets of a specific TF, *n* is the number of genes in the analyzed cluster, and *k* is the number of genes from that cluster that are annotated targets of the specific TF. We considered the gene cluster to be significantly enriched for TF targets at a *P*-value of *P* <0.001. Overrepresentation of MIPS functional categories was analyzed using FunSpec [61].

### Metabolome analyses

Cells were harvested by filtering 0.25 mL culture liquid on a polyamide membrane of 0.45 μm pore size (Sartorius). After washing the cells with 1 mL 0.5 g/L potassium acetate, the membrane was immediately transferred to a glass tube containing 5 mL of hot 75% ethanol and incubated for 4 min at 80°C before being placed on ice. (Acetate was added to the washing solution to minimize the impact of the washing step on intracellular metabolite concentrations by leaving the cells as along as possible in contact with the external carbon source.) Further sample processing and metabolite analysis by high-performance liquid chromatography have been described previously [62,63]. For metabolite quantification by LC-MS, a mixture of ^13^C fully labeled metabolites was added as an internal standard in all the samples [64,65]. This mixture consisted of extracts of yeast cells, cultivated on [U-^13^C_6_]-glucose and collected during both the exponential and the post-diauxic growth phases. LC-MS analyses and data processing were carried out as described previously [43]. Amino acids were measured using the AccQ-tag derivatization method from Waters™. Derivatized samples were analyzed on a high-performance liquid chromatograph equipped with a fluorescence detector (Dionex) as described [66]. To calculate intracellular metabolite concentrations, individual cells were assumed to have a volume of 82 μm^3^ [67].

### Enzymatic assays and cellular composition

Cells were harvested by filtering the culture medium on a polyamide membrane (0.45 μm pore size, Sartorius) and transferring the retained cells to liquid nitrogen using a spatula. Cell pellets were stored at -80°C until further analysis. Protocols for enzymatic assays are described in Additional files 2 and 15. Glycogen and trehalose content were assayed as described [68]. Protein content was measured with the method of Biuret.

### Western blotting

For analysis by immunoblot assays, cells were broken with glass beads (0.5 mm diameter) using a FastPrep-24 apparatus (MP Biomedicals) by eight successive pulses of 20 seconds at maximal speed, with one minute incubation on ice between pulses. The composition of the lysis buffer was 10 mM KCl, 5 mM MgCl_2_, 20 mM HEPES, pH 7.1, and also contained a protease inhibitor cocktail (cOmplete Mini EDTA-free, Roche). Cell extracts were then treated as described previously [69]. For each sample, 30 μg of total soluble proteins were separated by electrophoresis in 10% SDS polyacrylamide gels (BIORAD). Proteins were transferred to a nitrocellulose membrane, and blots were then cut in two halves, parallel to the migration front. The top parts of the membranes, corresponding to proteins with a molecular size above 75 kDa, were probed overnight at 4°C with rabbit polyclonal antibody (1:10,000; a kind gift from Prof. Jürgen Heinisch, Universität Osnabrück, Osnabrück, Germany) to detect Pfk1, while the lower parts of the same membranes were probed with mouse monoclonal anti-Pgk1 primary antibodies (Invitrogen, 1:2,000). Primary signals were then detected with either alkaline phosphatase-conjugated anti-rabbit secondary antibodies (for Pfk1) or alkaline phosphatase-conjugated anti-mouse secondary antibodies (for Pgk1), both used at a 1:20,000 dilution, and further revealed with chromogenic substrate solution BCIP/NBT (Sigma).

## Additional files

Additional file 1:
**Genes considered in the central metabolism.**
Additional file 2:
**Supplementary information regarding experimental procedures, metabolic flux estimations, and statistical methods for data analyses [** 6 **,** 70 **-** 86 **].**
Additional file 3: Figure S1. Identification of mid-stage repressed genes and their functional and regulatory analysis. **(A)** Partitioning around medoids clustering of 5,348 differentially expressed genes during meiotic development identified a cluster of 585 mid-repressed genes (cluster 6). **(B)** Functional classification of mid-repressed genes: the table shows enriched MIPS categories according to a hyper-geometric test applying a cutoff of *P* <0.001. **(C)** Transcription factor (TF) analysis of mid-repressed genes: predicted targets of 117 TFs [25] were intersected with mid-repressed genes in cluster 6. The table shows TFs whose targets were enriched among mid-repressed genes according to a hyper-geometric test applying a cutoff of *P* <0.001. TFs whose targets are also enriched among the 428 cluster 6 genes that have no ribosomal function are marked in bold-face. Additional file 4: Figure S2. Expression levels of the transcription factors Gcr1 and Gcr2 during meiotic development. Values are normalized to transcript abundance of the wild-type strain in YPA. Data represent the average of two independent experiments. The expression levels of *GCR1* and *GCR2* varied significantly between the SK1 *MAT* **a**/α and the SK1 *MAT*α/α strains at 6 h by four- and two-fold (with *P*-values of 0.029 and 0.012), respectively. Additional file 5: Figure S3. Statistical analysis of the correlations between enzymatic activities and expression levels of the corresponding genes. **(A)** Sporulating and **(B)** sporulation-deficient cells. Columns correspond to time points after transfer from YPA to sporulation medium. Each row corresponds to an enzymatic activity summarizing **(A)** Left panel: normalized ratios between enzymatic activities and the corresponding transcript levels (same as Figure 2), Right panel: significance levels of differences in these ratios between the reference condition (YPA) and the other time points. **(B)** Left panel: normalized ratio between enzymatic activity and the corresponding transcript levels. Second to the left panel: significance levels of differences in these ratios between the reference condition (YPA). Second to the right panel: normalized sum of the linearly scaled transcript levels. Right panel: normalized enzymatic activities. Additional file 6:
**Translation efficiencies of mid-stage repressed metabolic genes.**
Additional file 7: Figure S4. Statistical significance of differences in enzymatic activities between sporulating SK1 *MAT* ***a***/α cells, and sporulation-deficient SK1 *MATα*/α cells. Columns correspond to time points after transfer from YPA to sporulation medium. Each row corresponds to an enzymatic activity summarizing (left panel) the ratio between the enzymatic activity in sporulating and non-sporulating cells (same as Figure 3), (right panel) the significance levels of differences in enzymatic activities between sporulating and sporulation-deficient cells calculated for each time point in pairwise t-tests. Additional file 8:
**Measured enzymatic activities.**
Additional file 9: Figure S5. Concentrations of reserve carbohydrates in selected mutants after transfer to sporulation medium. Glycogen (left panel) and trehalose (right panel). Data represent the average of at least two independent experiments. Error bars show standard deviation. Additional file 10: Figure S6. Variation of metabolite concentrations in the sporulating SK1 *MAT* **a**/α and the sporulation-deficient SK1 *MAT*α/α strain during passage through meiosis or entry into starvation, respectively. The fold-change was calculated by dividing the highest by the lowest concentration of each metabolite measured over all time points. Additional file 11:
**Measured metabolite concentrations.**
Additional file 12: Figure S7. Statistical significance of differences of metabolite concentrations in sporulating SK1 *MAT* **a**/α cells and sporulation-deficient SK1 *MAT*α/α cells. Columns correspond to time points after transfer from YPA to sporulation medium. Each row corresponds to a metabolite concentration summarizing (left panel) the ratio of the concentrations in sporulating and non-sporulating cells (same as Figure 5), and (right panel) the significance levels of differences in metabolite concentrations between sporulating and sporulation-deficient cells calculated for each time point in pairwise t-tests. Additional file 13:
**Measured mass isotopologue concentrations.**
Additional file 14: Figure S8. Regulation of glutamate and nitrogen metabolism during meiotic development. **(A)** Activities of anabolic (Gdh1) and catabolic (Gdh2) glutamate dehydrogenase, and glutamate concentration in sporulating SK1 *MAT* **a**/α cells. **(B)** Protein content in sporulating SK1 *MAT* **a**/α and sporulation-deficient SK1 MATα/α control cells. **(C)** Concentration of 2-oxoglutarate, 2-oxoglutarate dehydrogenase (Kgd), and aconitase (Aco) in sporulating SK1 *MAT* **a**/α cells, all monitored after transfer to sporulation medium. Data represent the average of at least two independent experiments. Error bars show standard deviation. Additional file 15:
**Compositions of enzymatic assays employed in the study.**
