# Supplementary material for: Developmental stage-dependent metabolic regulation during meiotic differentiation in budding yeast
Source: BMC Biol. 2014 Sep 2;12:60. doi: 10.1186/s12915-014-0060-x (PMC4176597; doi:10.1186/s12915-014-0060-x)
Supplement: Additional file 2: — Supplementary information regarding experimental procedures, metabolic flux estimations, and statistical methods for data analyses [ 6 , 70 - 86 ]. [file 12915_2014_60_MOESM2_ESM.docx]

Content

[Supplementary Information S1: Strains and enzymatic assays 2](#_Toc387912536)

[Supplementary Information S2: Estimation of the fractions of acetate and glycogen-derived carbon in metabolites: 4](#_Toc387912537)

[Supplementary Information S3: Mass-isotopologue distribution in fructose-1,6bP and fructose-6P at 45 min after addition of ^13^C-labeled acetate 5](#_Toc387912538)

[Supplementary Information S4: Estimation of C-mol fraction of shikimate-derived amino acids in spore wall protein 5](#_Toc387912539)

[Supplementary Information S5: Relative contribution of acetate and glycogen-derived carbon to the formation of spore wall components 6](#_Toc387912540)

[Supplementary information S6: Statistical analysis of differences in activities, transcript levels, metabolite concentrations and composite measures 8](#_Toc387912541)

# Supplementary Information S1: Strains and enzymatic assays

**Table S1: Strains used in this study**

| **ID** | **Genotype** | **Reference/Origin** |
| --- | --- | --- |
| TWY35 | SK1 MATa/MATα ho::LYS2/ho::LYS2 ura3/ura3 lys2/lys2 leu2::hisG/leu2::hisG arg4-Nsp/arg4-Bgl his4x::LEU2-URA3/his4B::LEU2 | (Primig et al, 2000) [6] |
| TWY33 | SK1 MATα/MATα ho::LYS2/ho::LYS2 ura3/ura3 lys2/lys2 | (Primig et al, 2000) [6] |
| TWY189 | TWY35 + amd1::KanMX4/ amd1::KanMX4 | This study |
| TWY296 | TWY35 + isn1::KanMX4/ isn1::KanMX4 | This study |
| TWY56 | TWY35 + ume6::KanMX4/ ume6::KanMX4 | This study |
| TWY191 | TWY35 + sga1::KanMX4/ sga1::KanMX4 | This study |
| TWY225 | SK1 MATa/MATα ura3/ura3 his3/his3 CAN1/can1::Ste2::spHis5 flo8/flo8 | A. Deutschbauer (Stanford Genome Technology Center, Palo Alto, CA, USA) |
| TWY265 | TWY225 + ime2::kanMX4/ime2::kanMX4 | This study (*) |
| TWY241 | TWY225 + dmc1::kanMX4/dmc1::kanMX4 | This study (*) |
| TWY247 | TWY225 + smk1::kanMX4/smk1::kanMX4 | This study (*) |
| TWY233 | SK1 MATa/MATα ura3/ura3 his3/his3 CAN1/can1::Ste2::spHis5 flo8/flo8 ime1::kanMX4/ime1::kanMX4 | U. Schlecht (Stanford Genome Technology Center, Palo Alto, CA, USA) |
| TWY234 | SK1 MATa/MATα ura3/ura3 his3/his3 CAN1/can1::Ste2::spHis5 flo8/flo8 ndt80::kanMX/ndt80::kanMX | U. Schlecht (Stanford Genome Technology Center, Palo Alto, CA, USA) |

(*) Diploid strains carrying heterozygeous deletions of the corresponding genes in TWY225 were sporulated and segregants carrying the deletion were mated.

**Enzymatic assays**

Frozen cells were resuspended 0.5 mL breakage buffer (20 mM Hepes, 10 mM KCl, 5 mM MgCl_2_, pH 7.1) and transferred to thick-walled glass tubes containing 0.5 g glass beads (acid washed, 0.5 mm diameter, Sigma). Cells were broke open by vortexing (4 times for 20 s) and placing the samples on ice during the intervening time intervals to prevent heating. Crude extracts were centrifuged for 2 min at 4 °C at 12.000 x g and clear protein extract was retained. Protein concentration was estimated with the method of Bradford (Bradford, 1976) [71]. Protocols for the assay of hexokinase (Fromm, 1969) [74], glucose-6-phosphate dehydrogenase (Postma et al, 1989) [83], phosphoglucose isomerase (Van Hoek et al, 1998) [85], phosphofructose kinase (Bartrons et al, 1982) [70], fructose-1,6-bisphosphatase (Gancedo & Gancedo, 1971) [75], aldolase (Van Dijken et al, 1978) [84], glyceraldehyde-3-phosphate dehydrogenase, phosphoglycerate kinase and enolase (Van Hoek et al, 1998) [85], pyruvate kinase (Gancedo et al, 1967) [76], pyruvate decarboxylase (Postma et al, 1989) [83], phosphoenolpyruvate carboxykinase (Perea & Gancedo, 1982) [81], pyruvate carboxylase (Warren & Tipton, 1974) [86], alcohol dehydrogenase (Postma et al, 1989) [83], citrate synthase (Kim et al, 1936) [78], aconitase (Morrison, 1954) [80], isocitrate dehydrogenase (both, NAD and NADP-dependent) (Hirai et al, 1976) [77], malate synthetase (Dixon et al, 1960; Polakis & Bartley, 1965) [73, 82], isocitrate lyase (Polakis & Bartley, 1965) [82], malate dehydrogenase (Polakis & Bartley, 1965) [82], 2-oxoglutarate dehydrogenase (Carrillo-Castaneda & Ortega, 1970) [72], glutamate dehydrogenase (NAD and NADPH dependent) (Miller & Magasanik, 1990) [79], AcetylCoA synthase (Postma et al, 1989) [83] were adapted from the corresponding references. Enzymatic activities were measured at 30 °C at pH 7.1 in assay buffer containing 50 mM Hepes, 50 mM KCl, 5 mM MgCl_2_ and substrates and inhibitors specific for each reaction. The composition of each assay reaction is listed in Supplementary File 2. Production and consumption of NAD(P)H or 5',5'-dithiobis-(2-nitrobenzoate) (DTNB, Sigma) were monitored in a microplate reader (BioRad 680XR) at wavelengths of 340 nm or 415 nm, respectively. Enzymatic activities were estimated in at least two independent biological replicates each at two different dilutions of the protein extract to ensure that auxiliary enzymes are not rate-limiting. All auxiliary enzymes were purchased from Sigma.

# Supplementary Information S2: Estimation of the fractions of acetate and glycogen-derived carbon in metabolites:

The fraction of acetate-derived carbon (F_a_) in each metabolite pool was estimated assuming that a label abundance of *A* = 0.57, which equals the label abundance of acetate in the medium, indicates a F_a_ of 1. The fraction of acetate-derived carbon in glyceraldehyde-3P and DHAP was assumed to be the same as in fructose-1,6bP (0.88) since aldolase was highly active throughout meiotic differentiation. The F_a_ of glucan, chitosan, and mannan was assumed to equal their corresponding precursors glucose-6P (0.46) and fructose-6P (0.53). The concentrations of ribose-5P, ribulose-5P and xylulose-5P could not be quantified individually. These sugars were measured as a single metabolite pool denoted pentose-5P. Measured F_a_ of the pentose-5P pool was 0.56. The F_a_ of ribose-5P was inferred from PRPP (0.54). F_a_  in erythrose-4P and xylulose-5P was estimated according to equation system below (Equations S1 - S3) assuming that there is significant exchange of carbon via the transaldolase (TA) and both transketolase (TK1 and TK2) reactions.

TK1: n_S7P_ F_a,S7P_ + n_GAP_ F_a,GAP_ = n_R5P_ F_a,R5P_ + n_X5P_ F_a,X5P_ (S1)

TK2: n_F6P_ F_a,F6P_ + n_GAP_ F_a,GAP_ = n_E4P_ F_a,E4P_ + n_X5P_ F_a,X5P_ (S2)

TA: n_S7P_ F_a,S7P_ + n_GAP_ F_a,GAP_ = n_E4P_ F_a,E4P_ + n_F6P_ F_a,F6P_ (S3)

(n is the number of carbon atoms in the metabolite)

The equation system was overdetermined and yielded 0.63 and 0.6 as the best approximations for the fraction of acetate-derived carbon in erythrose-4P and xylulose-5P, respectively. The F_a,Shi3P_ of shikimate-3P was calculated according to Equation S4 to be 0.77.

n_PEP_ F_a,PEP_ + n_E4P_ F_aE4P_ = n_Shi3P_ F_a,Shi3P_ (S4)

# Supplementary Information S3: Mass-isotopologue distribution in fructose-1,6bP and fructose-6P at 45 min after addition of ^13^C-labeled acetate


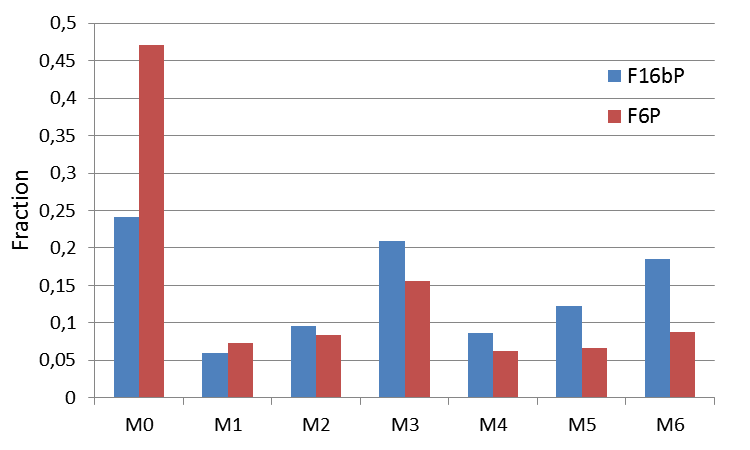


Figure S5: Comparison of mass-isotopologue distribution in fructose-1,6bP and fructose-6P at 45 min after addition of ^13^C-labeled acetate

# Supplementary Information S4: Estimation of C-mol fraction of shikimate-derived amino acids in spore wall protein

The mole fractions of all amino acids in the spore wall protein were reported by Brisza *et al.* 1986 (Table S2). Using these values, the C-mol fraction (*p_Cmol,prot,i_*) of each amino acid (*i*) in the spore wall protein was calculated according to Equation S5

$p_{Cmol,prot,i}=\frac{p_{mol,prot,i}\cdot n_{i}}{\sum_{i} p_{mol,prot,i}\cdot n_{i}}\cdot100$ (S5)

wherein *p_mol,prot,i_* is the mole fraction of an amino acid in the spore wall protein, and *n* is the number of C-atoms in this amino acid. The mass fraction *p_m,prot,i_* of each amino acid in the spore wall protein was calculated according to Equation S6

$p_{m,prot,i}=\frac{p_{mol,i}\cdot M_{i}}{\sum_{i} p_{mol,i}\cdot M_{i}}\cdot100$ (S6)

wherein *M_i_* is the molar mass of the amino acid. The results are listed in Table S2. The mass fraction of shikimate-derived amino acids tyrosine, dityrosine, and phenylalanine in the spore wall protein adds up to 0.52 which corresponds to a C-mol fraction of 0.6.

Table S2: Estimation of C-mol fractions of amino acids in spore wall protein

| **Amino acid** | **M [g/mol]** | **C-atoms in AA** | **Mole* fraction** |  | **C-mol fraction**  (*p_Cmol,prot,i_*) | **mass fraction**  (*p_m,prot,i_*) |
| --- | --- | --- | --- | --- | --- | --- |
| ser | 105 | 3 | 0,025 |  | 0,01 | 0,02 |
| asp | 133 | 4 | 0,06 |  | 0,03 | 0,05 |
| glu | 147 | 5 | 0,037 |  | 0,02 | 0,03 |
| arg | 174 | 6 | 0,005 |  | 0,00 | 0,01 |
| thr | 119 | 4 | 0,009 |  | 0,00 | 0,01 |
| gly | 75 | 2 | 0,15 |  | 0,04 | 0,07 |
| ala | 89 | 3 | 0,059 |  | 0,02 | 0,03 |
| met | 149 | 5 | 0,004 |  | 0,00 | 0,00 |
| pro | 115 | 5 | 0,063 |  | 0,04 | 0,04 |
| val | 117 | 5 | 0,043 |  | 0,03 | 0,03 |
| phe | 165 | 9 | 0,028 |  | **0,03** | **0,03** |
| leu | 131 | 6 | 0,061 |  | 0,05 | 0,05 |
| ile | 131 | 6 | 0,055 |  | 0,04 | 0,04 |
| lys | 146 | 6 | 0,127 |  | 0,10 | 0,11 |
| his | 155 | 6 | 0 |  | 0,00 | 0,00 |
| dityr | 360 | 18 | 0,206 |  | **0,49** | **0,43** |
| tyr | 182 | 9 | 0,059 |  | **0,07** | **0,06** |
| SUM |  |  | 1 |  | 1 | 1 |

(*) Data from Brisza et al./1986/JBC/261/4288-4294)

# Supplementary Information S5: Relative contribution of acetate and glycogen-derived carbon to the formation of spore wall components

The mass fractions of the spore wall components glucan, mannan, chitosan, and protein were reported to be 0.55, 0.17, 0.09, and 0.12, respectively (Brisza *et al.* 1986). These mass fractions only add up to only 0.93 which may indicate small experimental errors, or presence of unknown compounds. We neglected this error of 7 % by scaling the reported mass fractions to unity. Thus, the values 0.59, 0.18, 0.1, and 0.13 were used as the mass fractions of glucan, mannan, chitosan, and protein, respectively, in our calculations. To further simplify the model, we only took into account the shikimate-derived amino acid fraction of the spore wall protein (in the following named dityrosine) since these amino acids account for more than half of this protein (Table S2). Thus, the error introduced by these model simplifications adds up to a maximum of 14 %.

The C-Mol fractions (*p_Cmol,sw,j_*) of the spore wall components (*j*) were calculated using Equation S7

$p_{Cmol,sw,j}=\frac{\frac{p_{m,sw,j}}{M_{j}}\cdot n_{j}}{\sum_{j} \frac{p_{m,sw,j}\cdot n_{j}}{M_{j}}}$ (S7)

wherein *p_m,sw_* is the mass fraction of the spore wall component, *M* is the molar mass of the corresponding monomer, and *n* is the number of carbon atoms of the monomer. The results are listed in Table S3.

Table S3: Estimation of the C-Mol fraction of each spore wall component

|  |  |  |  |  |  |  |
| --- | --- | --- | --- | --- | --- | --- |
| **Spore wall component** | **C-atoms** | **M [g/mol]** | **Mass* fraction**  (*p_m,sw_*) | **mol/g_SW_** | **C-Mol/g_SW_** | **C-Mol fraction**  (*p_Cmol,sw,j_*) |
| glucan | 6 | 180 | 0,59 | 0,00328 | 0,020 | **0,61** |
| chitosan | 6 | 179 | 0,10 | 0,00056 | 0,003 | **0,10** |
| mannan | 6 | 180 | 0,18 | 0,00100 | 0,006 | **0,19** |
| dityrosine | 18 | 360 | 0,07** | 0,00019 | 0,003 | **0,10** |
| SUM |  |  | 0,93 | 0,005024 | 0,032 | **1,0** |

(*) Data from Brisza et al./1986/JBC/261/4288-4294 normalized to 100 %, (**) assuming that the mass fraction of shikimate-derived amino acids in the spore wall protein equals 0.52 (see Table S2).

The relative contribution of the carbon sources acetate and glycogen to the formation of the spore wall components glucan, chitosan, mannan, and dityrosine was calculated by multiplying the C-mol fraction of each spore wall component (*p_Cmol,sw,j_*) with the fractions of acetate (F_a_) and glycogen-derived (F_g_) carbon in the corresponding precursor (Table S4). The results are listed in Table S4.

Table S4: Estimation of the relative contribution of acetate and glycogen-derived carbon to the formation of each spore wall component.

| **Spore wall**  **Component** | **C-Mol fraction**  (*p_Cmol,sw,j_*) | **Precursor** | **Fractions of acetate and glycogen-derived carbon in precursor** | | **Fraction of carbon derived from acetate or glycogen in spore wall component normalized to total carbon in spore wall** | |
| --- | --- | --- | --- | --- | --- | --- |
|  |  |  | **F_g_** | **F_a_** | **glycogen** | **acetate** |
| glucan | 0,61 | glucose-6P | 0,59 | 0,41 | **0,36** | **0,25** |
| chitosan | 0,10 | fructose-6P | 0,54 | 0,46 | **0,06** | **0,05** |
| mannan | 0,19 | fructose-6P | 0,54 | 0,46 | **0,10** | **0,09** |
| dityrosine | 0,10 | shikimate-3P | 0,23 | 0,77 | **0,02** | **0,08** |
| **SUM** | 1,0 |  |  |  | **0,54** | **0,46** |

# Supplementary information S6: Statistical analysis of differences in activities, transcript levels, metabolite concentrations and composite measures

In the first step it was analyzed whether enzyme activities, metabolome, and transcriptome measurements had the same average (intra-dataset) variance (*s*) at all time points *t* (including YPA). *s* was calculated according to Equation S8 wherein *r_i_* are the values of the replicates, $\bar{r}$ is their average, and *z* is the number of individuals in the dataset (e.g. the number of metabolites).

$s(t)=\frac{\sum_{z} \left( \frac{\sqrt{\sum_{i=1}^{2} {(r_{i}\left( t \right)-\bar{r}\left( t \right))}^{2}}}{\bar{r}(t)} \right)}{z}$ (S8)

Comparison of these variations in the data showed that measurements of enzyme activities, metabolite concentrations, and transcript levels had the same (intra-dataset) variance over the whole time course (= differences were not statistically significant, data not shown). Having this established, the standard deviation for the measurement of an individual activity, transcript level, or metabolite concentration was estimated at the time point where most replicate measurements were available. Since the SK1 *MAT***a**/α and the SK1 *MAT*α/α strains have the same behavior under growth conditions, we therefore chose the YPA time point to calculate the standard deviation of our measurements from the pooled replicates of the two strains (two replicates each = 4 in total). This standard deviation (calculated for each individual gene, activity, or metabolite) was used to perform pairwise t-tests at all time points. The average relative standard deviations for the estimations of enzymatic activities, transcript levels, and metabolite concentrations were 16 %, 8.5 %, and 24 %, respectively.

The ratio (*R*) at time *t* of the activity of an enzyme over the sum of expression levels of the genes that encode the enzymatic activity was calculated according to Equation S9.

$R(t)=\frac{\bar{a}(t)}{\sum_{j=1}^{k} \bar{L}_{j}(t)}$ (S9)

wherein $\bar{a}$ and $\bar{L}$ are the average activities and expression levels, respectively, calculated from the replicates at a given time point, and *k* is the number of genes that encode the activity. The relative standard deviation (*s_L_*) of the sum of transcript levels, and the relative standard deviation of the activity measurements (*s_a_*) were calculated according to Equations S10, and S11, respectively, wherein *n* is the number of replicates and *k* is the number of genes encoding the activity.

$s_{L}=\frac{\sqrt{\sum_{j=1}^{k} \left( \frac{\sum_{i=1}^{n} {(L_{ij}-\bar{L}_{j})}^{2}}{n-1} \right)}}{\sum_{j=1}^{k} \bar{L}_{j}}$ (S10)

$s_{a}=\frac{\sqrt{\frac{\sum_{i=1}^{n} {(a_{i}-\bar{a)}}^{2}}{n-1}}}{\bar{a}}$ (S11)

The relative standard deviation (*s_R_*) in the estimations of *R* was calculated by equation

${s_{R}}^{2}={s_{L}}^{2}+{s_{a}}^{2}$. (S12)

It was estimated at the YPA time point for each individual enzyme by pooling measurements of activities and transcript levels of the SK1 *MAT***a**/α and the SK1 *MAT*α/α strains. The corresponding standard deviation *s_r,YPA_* (Equation S13)

$s_{r,YPA}=s_{R,YPA}\cdot R_{YPA}$ (S13)

was used to compare *R*-values between YPA and different time points during sporulation in pairwise t-tests. The average relative standard deviation of *R* was 19.3 %.
