# Supplementary figures and images for: Developmental stage-dependent metabolic regulation during meiotic differentiation in budding yeast
Source: BMC Biol. 2014 Sep 2;12:60. doi: 10.1186/s12915-014-0060-x (PMC4176597; doi:10.1186/s12915-014-0060-x)

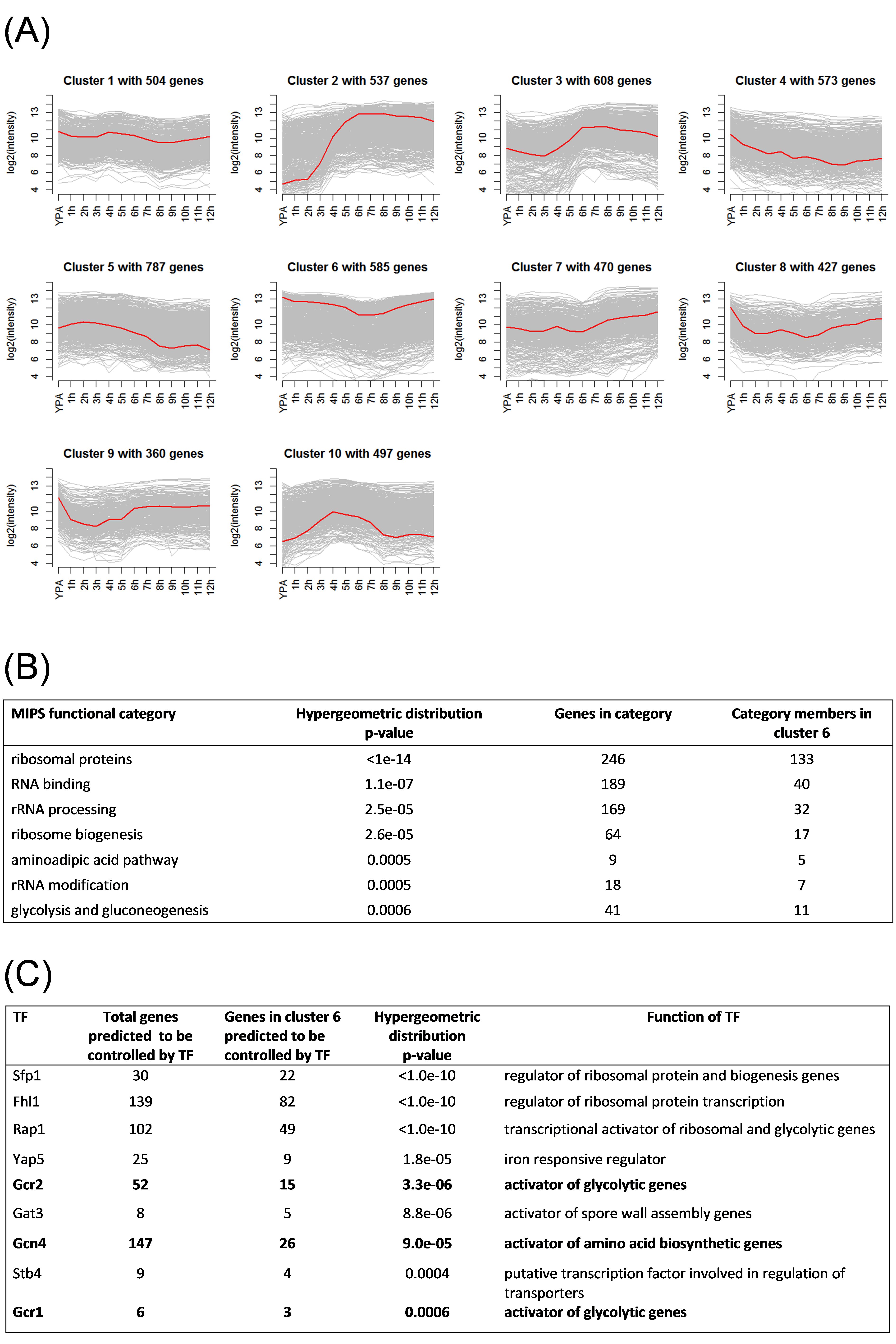

Supplement: Additional file 3: Figure S1. — Identification of mid-stage repressed genes and their functional and regulatory analysis. (A) Partitioning around medoids clustering of 5,348 differentially expressed genes during meiotic development identified a cluster of 585 mid-repressed genes (cluster 6). (B) Functional classification of mid-repressed genes: the table shows enriched MIPS categories according to a hyper-geometric test applying a cutoff of P <0.001. (C) Transcription factor (TF) analysis of mid-repressed genes: predicted targets of 117 TFs [25] were intersected with mid-repressed genes in cluster 6. The table shows TFs whose targets were enriched among mid-repressed genes according to a hyper-geometric test applying a cutoff of P <0.001. TFs whose targets are also enriched among the 428 cluster 6 genes that have no ribosomal function are marked in bold-face. [file 12915_2014_60_MOESM3_ESM.jpeg]

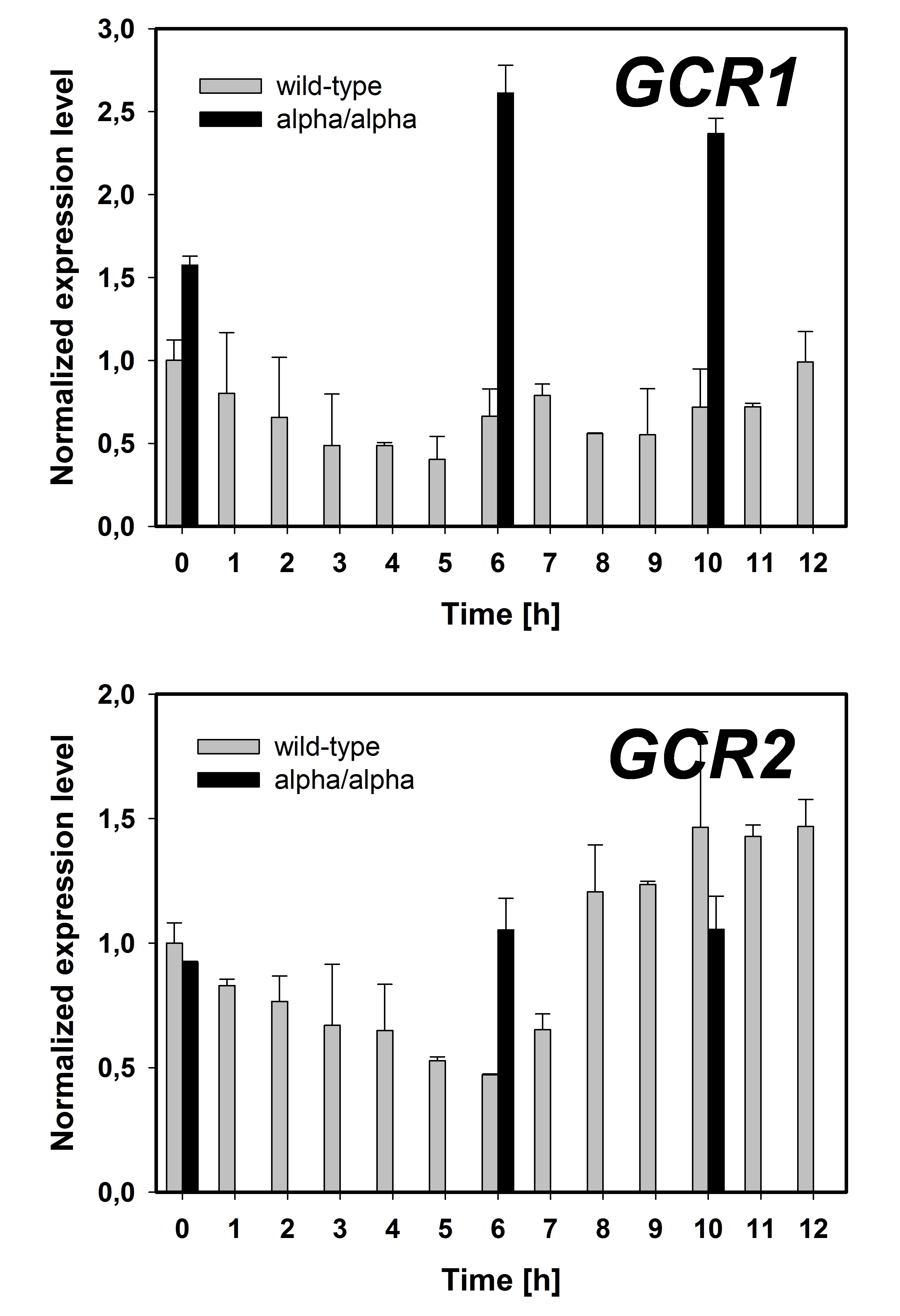

Supplement: Additional file 4: Figure S2. — Expression levels of the transcription factors Gcr1 and Gcr2 during meiotic development. Values are normalized to transcript abundance of the wild-type strain in YPA. Data represent the average of two independent experiments. The expression levels of GCR1 and GCR2 varied significantly between the SK1 MAT a/α and the SK1 MATα/α strains at 6 h by four- and two-fold (with P-values of 0.029 and 0.012), respectively. [file 12915_2014_60_MOESM4_ESM.jpeg]

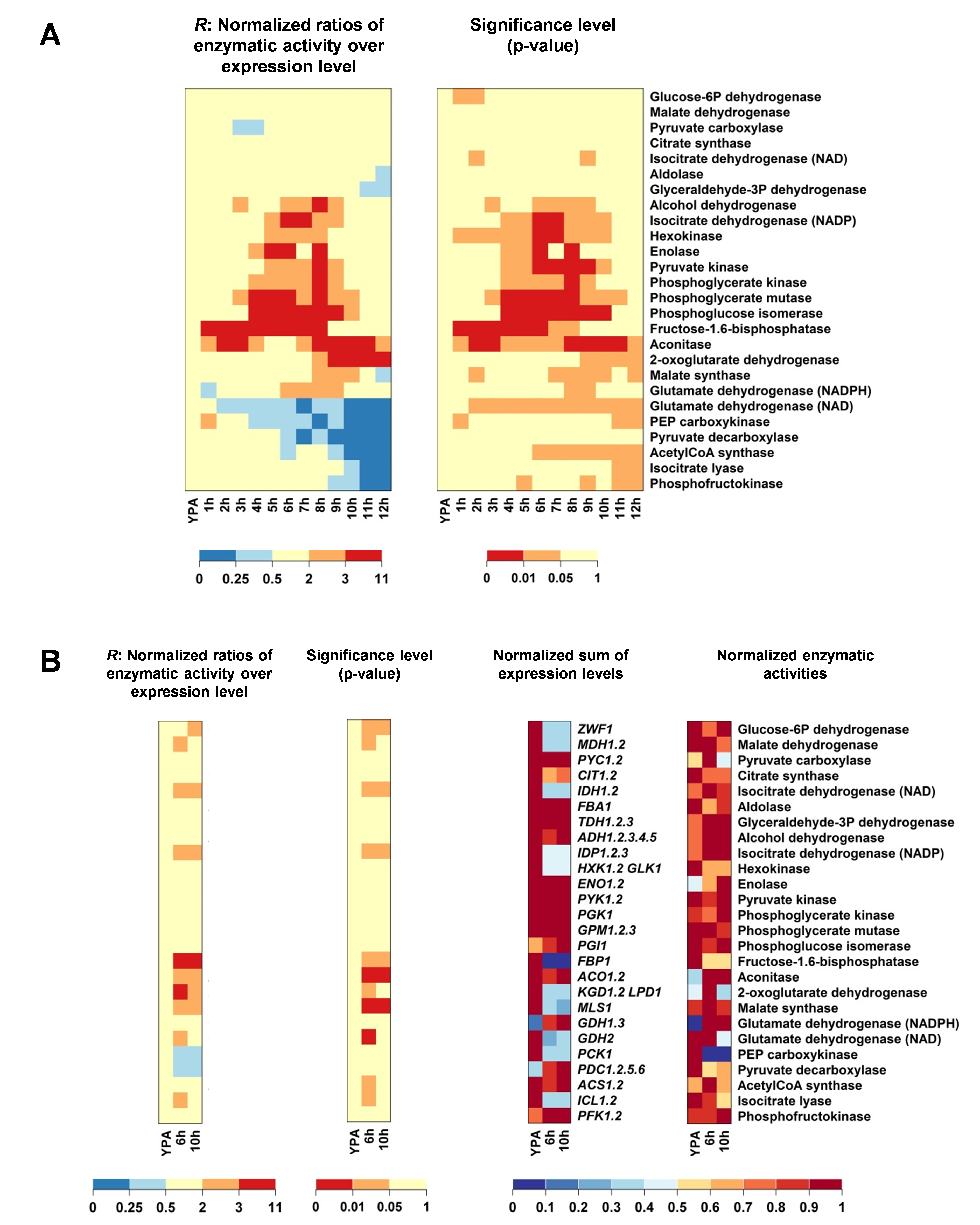

Supplement: Additional file 5: Figure S3. — Statistical analysis of the correlations between enzymatic activities and expression levels of the corresponding genes. (A) Sporulating and (B) sporulation-deficient cells. Columns correspond to time points after transfer from YPA to sporulation medium. Each row corresponds to an enzymatic activity summarizing (A) Left panel: normalized ratios between enzymatic activities and the corresponding transcript levels (same as Figure 2), Right panel: significance levels of differences in these ratios between the reference condition (YPA) and the other time points. (B) Left panel: normalized ratio between enzymatic activity and the corresponding transcript levels. Second to the left panel: significance levels of differences in these ratios between the reference condition (YPA). Second to the right panel: normalized sum of the linearly scaled transcript levels. Right panel: normalized enzymatic activities. [file 12915_2014_60_MOESM5_ESM.jpeg]

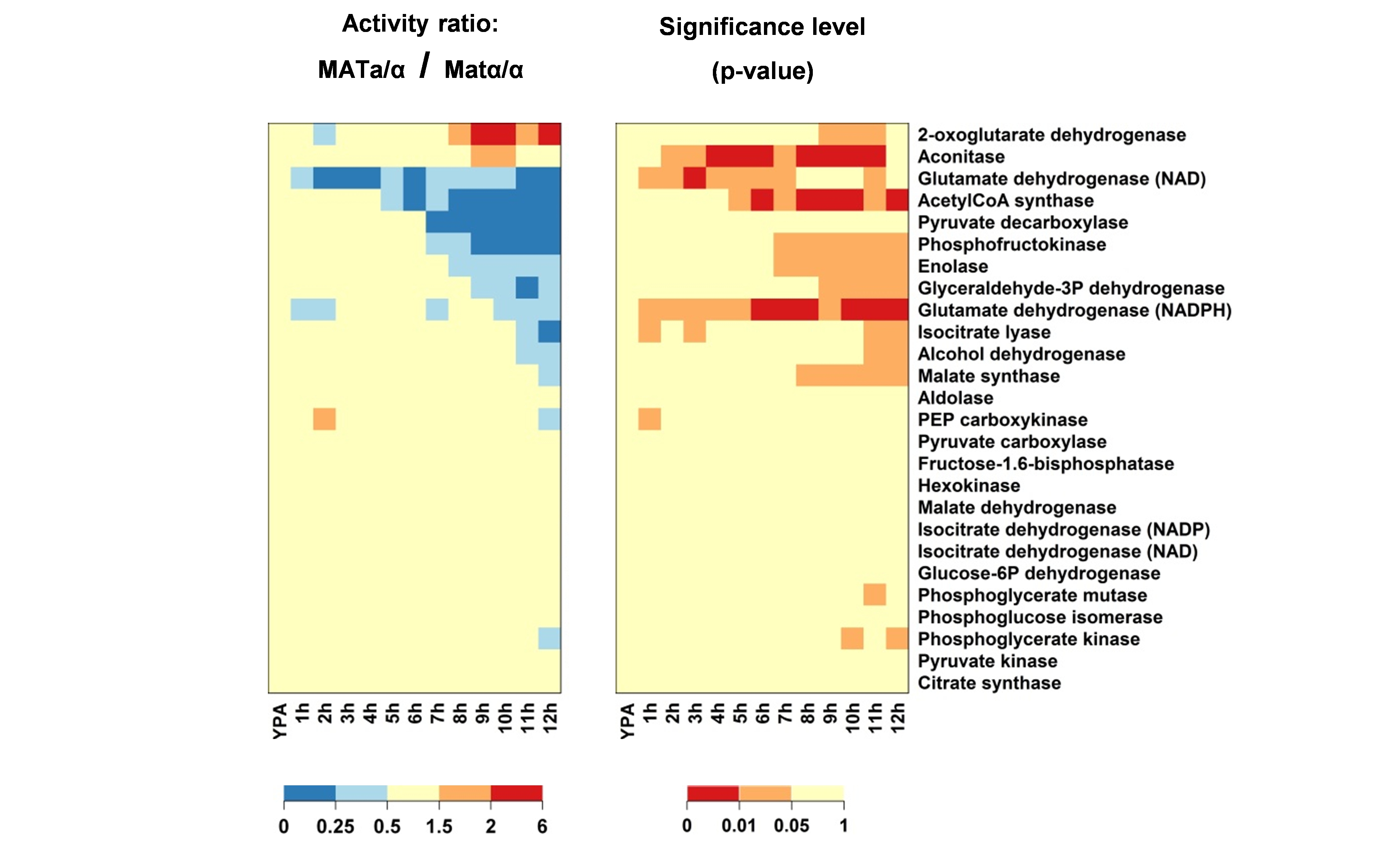

Supplement: Additional file 7: Figure S4. — Statistical significance of differences in enzymatic activities between sporulating SK1 MAT a/α cells, and sporulation-deficient SK1 MATα/α cells. Columns correspond to time points after transfer from YPA to sporulation medium. Each row corresponds to an enzymatic activity summarizing (left panel) the ratio between the enzymatic activity in sporulating and non-sporulating cells (same as Figure 3), (right panel) the significance levels of differences in enzymatic activities between sporulating and sporulation-deficient cells calculated for each time point in pairwise t-tests. [file 12915_2014_60_MOESM7_ESM.jpeg]

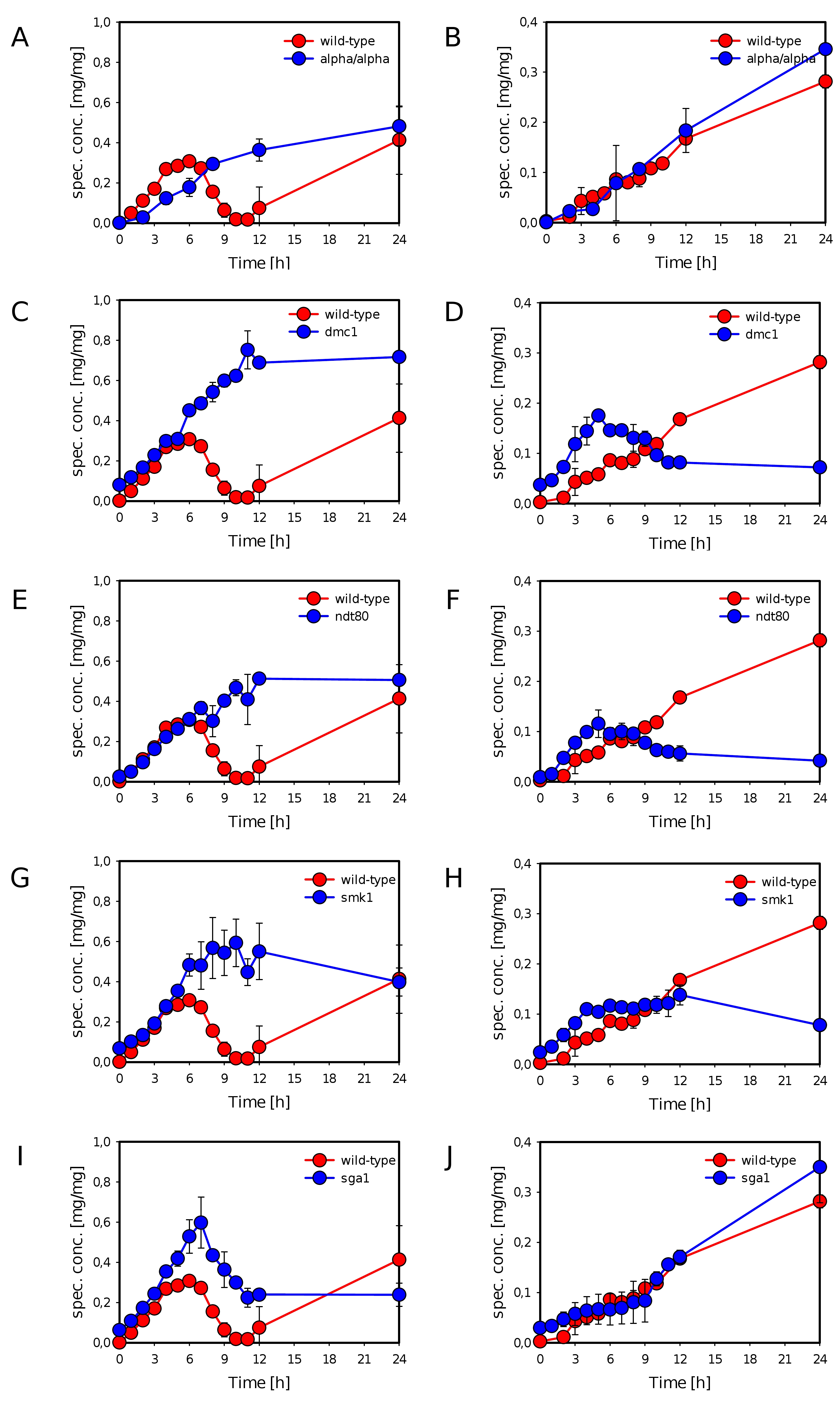

Supplement: Additional file 9: Figure S5. — Concentrations of reserve carbohydrates in selected mutants after transfer to sporulation medium. Glycogen (left panel) and trehalose (right panel). Data represent the average of at least two independent experiments. Error bars show standard deviation. [file 12915_2014_60_MOESM9_ESM.jpeg]

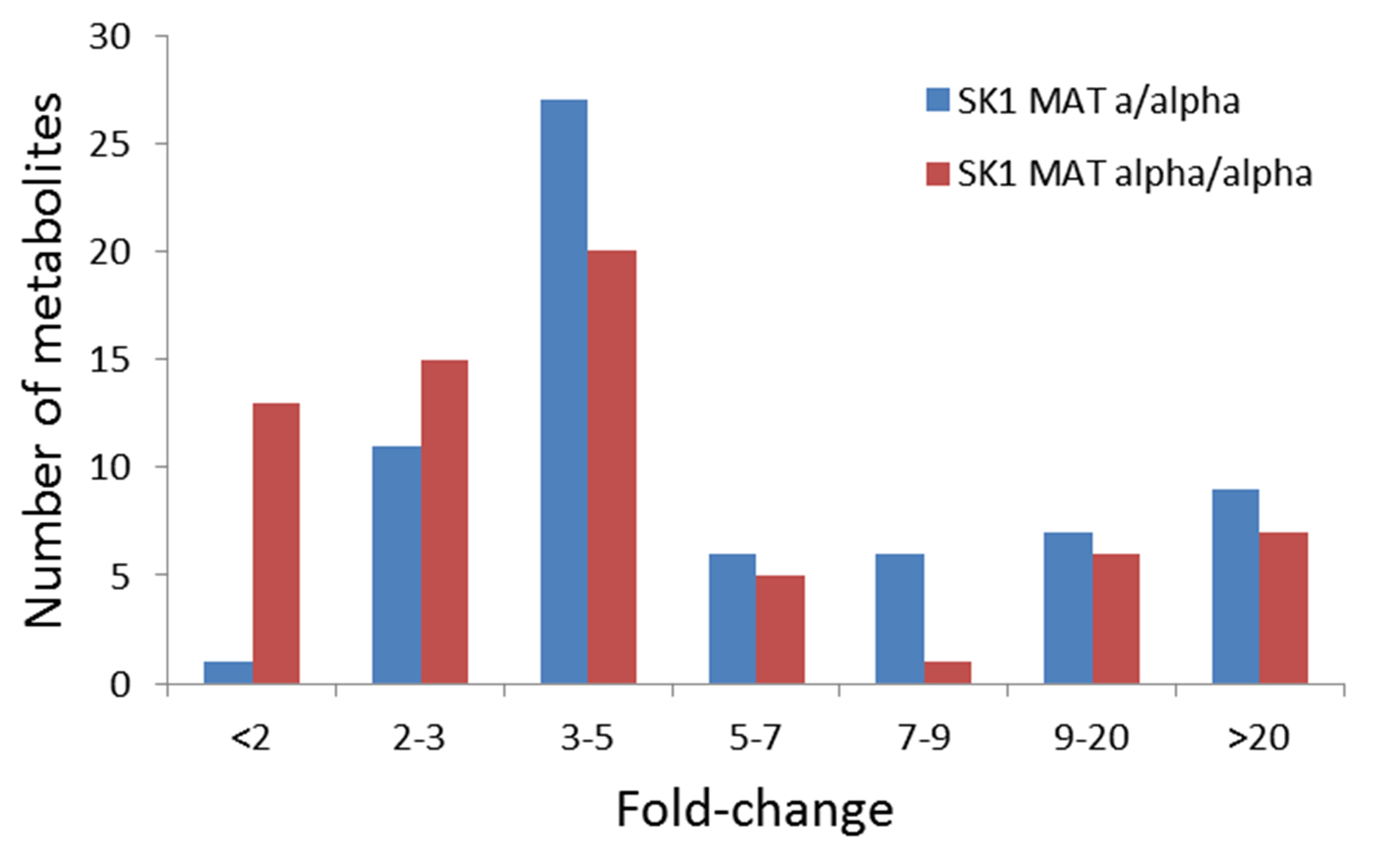

Supplement: Additional file 10: Figure S6. — Variation of metabolite concentrations in the sporulating SK1 MAT a/α and the sporulation-deficient SK1 MATα/α strain during passage through meiosis or entry into starvation, respectively. The fold-change was calculated by dividing the highest by the lowest concentration of each metabolite measured over all time points. [file 12915_2014_60_MOESM10_ESM.jpeg]

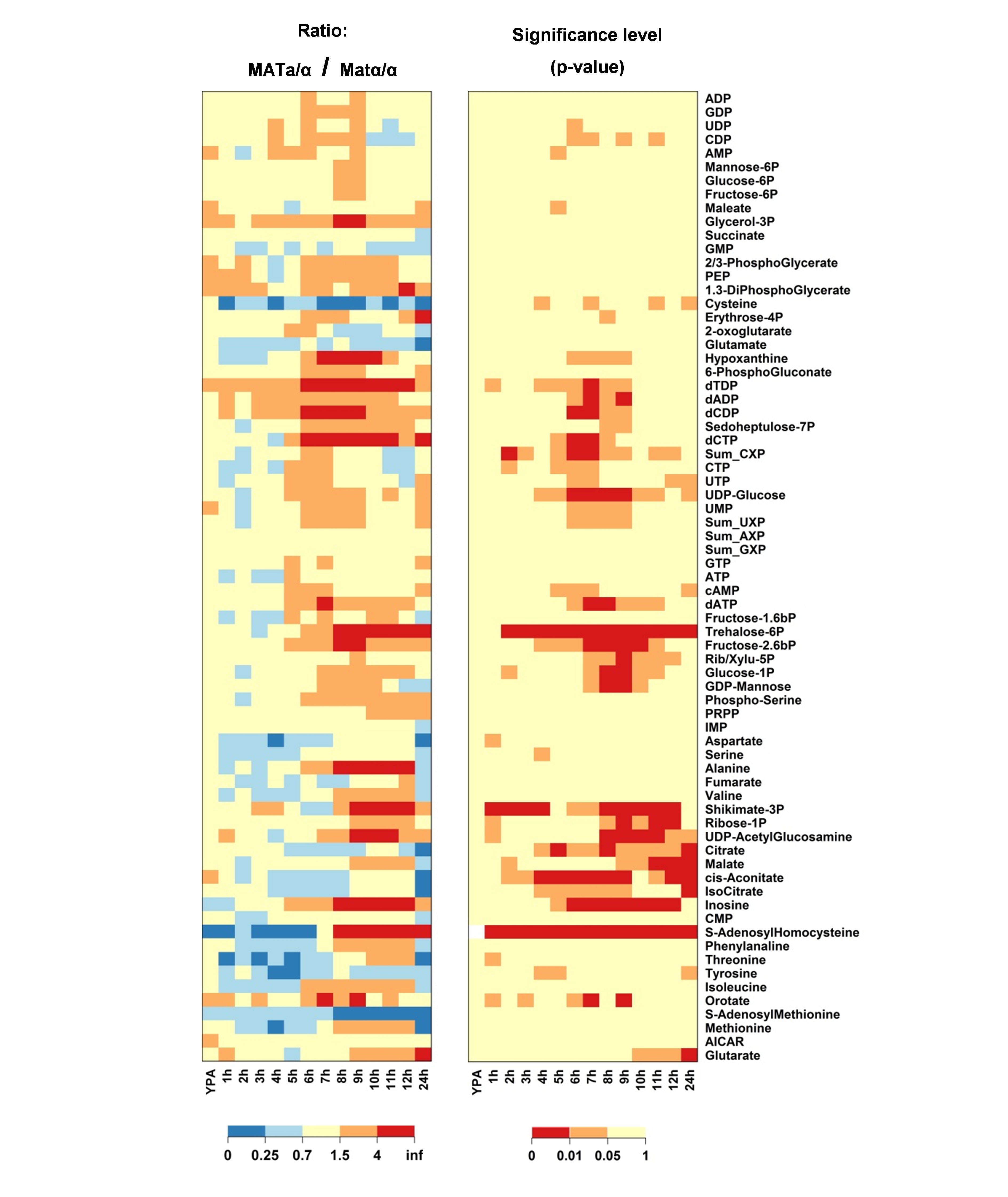

Supplement: Additional file 12: Figure S7. — Statistical significance of differences of metabolite concentrations in sporulating SK1 MAT a/α cells and sporulation-deficient SK1 MATα/α cells. Columns correspond to time points after transfer from YPA to sporulation medium. Each row corresponds to a metabolite concentration summarizing (left panel) the ratio of the concentrations in sporulating and non-sporulating cells (same as Figure 5), and (right panel) the significance levels of differences in metabolite concentrations between sporulating and sporulation-deficient cells calculated for each time point in pairwise t-tests. [file 12915_2014_60_MOESM12_ESM.jpeg]

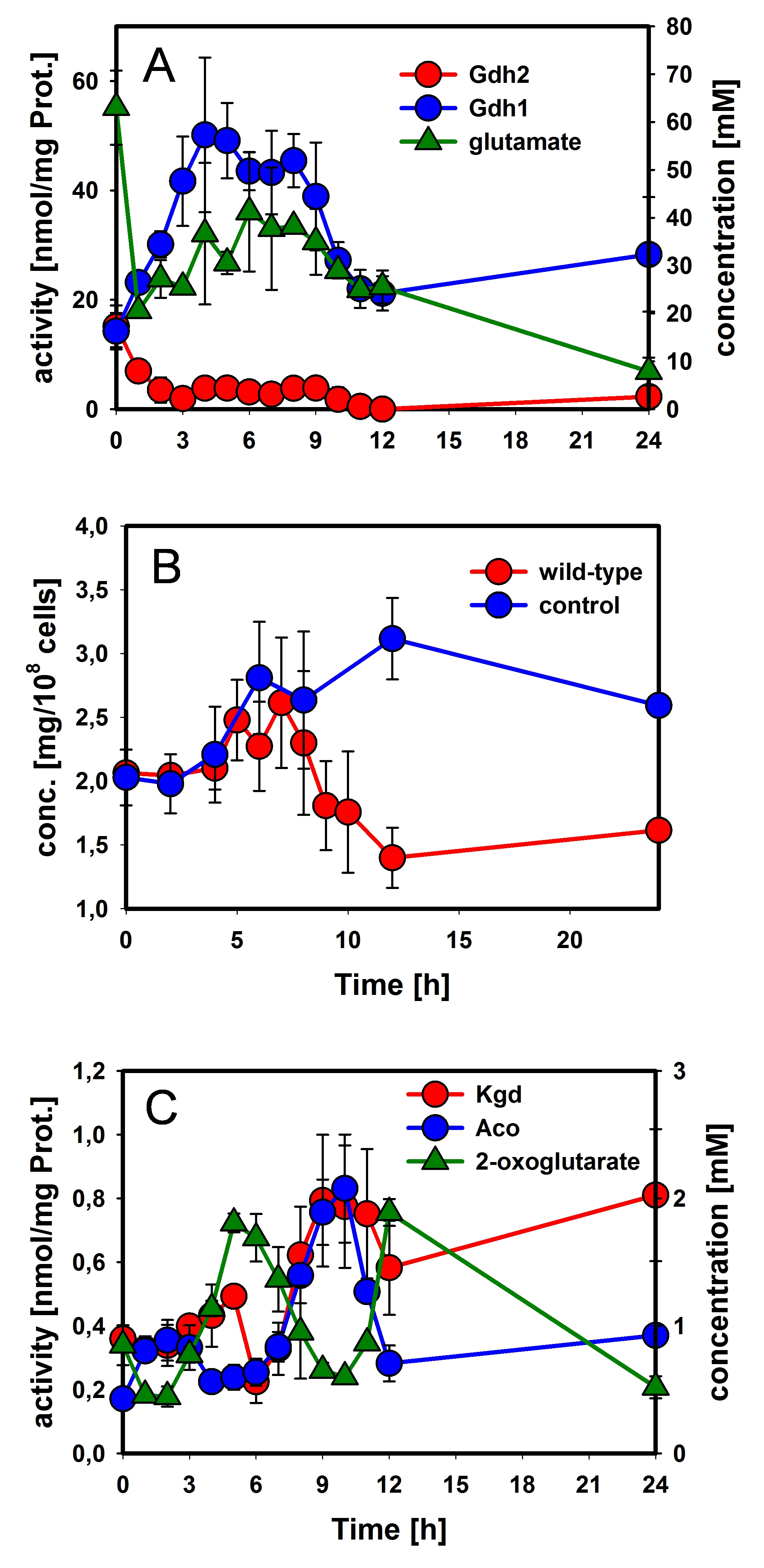

Supplement: Additional file 14: Figure S8. — Regulation of glutamate and nitrogen metabolism during meiotic development. (A) Activities of anabolic (Gdh1) and catabolic (Gdh2) glutamate dehydrogenase, and glutamate concentration in sporulating SK1 MAT a/α cells. (B) Protein content in sporulating SK1 MAT a/α and sporulation-deficient SK1 MATα/α control cells. (C) Concentration of 2-oxoglutarate, 2-oxoglutarate dehydrogenase (Kgd), and aconitase (Aco) in sporulating SK1 MAT a/α cells, all monitored after transfer to sporulation medium. Data represent the average of at least two independent experiments. Error bars show standard deviation. [file 12915_2014_60_MOESM14_ESM.jpeg]
